# The synergistic action of reduced membrane permeability and antibiotic sequestration as a novel mechanism for carbapenem resistance in *Campylobacter*

**DOI:** 10.1128/mbio.00364-26

**Published:** 2026-05-20

**Authors:** Xinggui Chen, Yulian Zhou, Quan Zhou, Min He, Mengyu Zhao, Suchuan Dai, Jinpeng Li, Chengyao Hou, Weilai Tao, Xinyi Yang, Shiying Lan, Xin Yang, Changwei Lei, Anyun Zhang, Hongning Wang, Yizhi Tang

**Affiliations:** 1 Key Laboratory of Bioresource and Eco-environment of the Ministry of Education, College of Life Sciences, Sichuan University 162677, Chengdu, China; 2 Animal Disease Prevention and Green Development Key Laboratory of Sichuan Province, College of Life Sciences, Sichuan University 162677, Chengdu, China; Ludwig-Maximilians-Universitat Munchen, Munich, Germany

**Keywords:** *Campylobacter*, carbapenem resistance, outer membrane proteins, OXA-61, synergism

## Abstract

Carbapenems are last-resort antibiotics against gram-negative pathogens, including multidrug-resistant *Campylobacter*. However, carbapenem-resistant *Campylobacter* strains are emerging and have been isolated in patients treated with carbapenems. This emerging resistance mechanism may be underpinned by multiple genetic determinants, yet it has not been systematically characterized or elucidated until now. In this study, two candidate mutations identified via resistance evolution and whole-genome sequencing were validated through genetic and functional assays. *De novo* structural modeling and molecular dynamics simulations elucidated the impact of mutations on protein dynamics. Furthermore, carbapenem hydrolysis and binding-pocket interactions were characterized using ultra-high-performance liquid chromatography, MM/PBSA calculations, and alanine substitution experiments. Our findings reveal that carbapenem resistance in *Campylobacter* is mediated by synergistic mutations in *porA*, encoding the major outer membrane protein (MOMP), and *bla*_OXA-61_, encoding a non-carbapenemase β-lactamase OXA-61. Specifically, the D157H substitution in MOMP induces structural and functional remodeling, thereby hindering meropenem translocation across the outer membrane, while a G → T transversion in the *bla*_OXA-61_ promoter leads to overexpression of the enzyme that sequesters meropenem instead of hydrolyzing it. Notably, either *porA* alteration or *bla*_OXA-61_ overexpression alone confers only a modest increase in carbapenem resistance (2- to 8-fold), whereas their synergy yields a high-level resistance (16- to 128-fold). Together, these results define a novel carbapenem resistance mechanism mediated by reduced porin permeability coupled with antibiotic sequestration acted by a non-carbapenemase β-lactamase. This synergistic mechanism explains clinical carbapenem resistance phenotypes in *Campylobacter*, facilitates diagnosis and surveillance, and likely represents a general carbapenem resistance strategy across other bacterial species.

**IMPORTANCE:**

*Campylobacter* ranks among the leading causes of bacterial gastroenteritis worldwide. In recent years, carbapenem-resistant *Campylobacter* strains have been emerging in clinical settings and have been increasingly reported from patients following carbapenem treatment. Despite the importance of carbapenems in therapeutic treatment of multidrug-resistant *Campylobacter*, the molecular basis of this resistance phenotype remains poorly understood. Additionally, non-carbapenemase β-lactamases have been implicated in carbapenem resistance in many bacterial species, but how they contribute to the resistance without hydrolyzing the antibiotic is unknown. Our study defines a new mechanism for carbapenem resistance and accounts for the carbapenem-resistant phenotype observed in clinical isolates. They also provide timely information useful for the diagnosis and treatment of infections caused by antibiotic-resistant *Campylobacter*. Equally important, this study provides a mechanistic explanation for carbapenem resistance mediated by non-carbapenemase β-lactamases, which exist in many gram-negative pathogens.

## INTRODUCTION

*Campylobacter* species, notably *Campylobacter jejuni* (*C. jejuni*) and *Campylobacter coli* (*C. coli*), rank among the leading causes of bacterial gastroenteritis worldwide (1). In the United States alone, the Centers for Disease Control and Prevention estimates approximately 1.5 million cases of human campylobacteriosis occur annually (2). In Europe, *Campylobacter* is the most common cause of bacterial enteritis and the leading cause of foodborne illness (2). Although most *Campylobacter* infections are self-limiting and typically resolve within several days without antimicrobial therapy (3), certain groups, including the young, elderly, and immunocompromised individuals, are at higher risk of severe or persistent illness (4). In such cases, fluoroquinolones and macrolides remain the first-line antibiotics (5). Carbapenems, such as meropenem, are generally reserved for severe systemic infections like bacteremia, particularly when isolates show resistance to both fluoroquinolones and macrolides (6). Previously, carbapenem resistance in *Campylobacter* was rare; however, carbapenem-resistant *Campylobacter* strains are emerging and have been increasingly reported from patients following carbapenem treatment (5, 7–9).

Carbapenems are critically important antibiotics, as they are often considered the last-resort treatment for serious infections (5, 7). The best-known mechanism of carbapenem resistance is antibiotic hydrolysis mediated by carbapenemases, which are mainly plasmid-encoded and highly transmissible (10, 11). Other non-carbapenemase-mediated carbapenem resistance mechanisms include overexpression of antibiotic efflux pumps, reduced membrane permeability, and production of β-lactamases such as ESBL and AmpC β-lactamase (12). Although non-carbapenem hydrolyzing β-lactamases are involved in carbapenem resistance (13), the underlying mechanisms remain unknown.

To date, no classical carbapenemases have been identified in *Campylobacter*, suggesting the existence of a novel carbapenem resistance mechanism in this non-carbapenemase-producing species. A previous study hypothesized a potential association between mutations in the major outer membrane protein (MOMP) and carbapenem resistance in *Campylobacter* (5). However, the role of MOMP in carbapenem resistance has not been formally defined. The MOMP is known to serve multiple physiological functions and constitutes the main pore through which antibiotics enter *Campylobacter* cells (14, 15). Although little sequence homology exists with other porin proteins, MOMP shares structural and functional similarities with OmpC and OmpF of *Escherichia coli* (*E. coli*). Unlike *E. coli*, *Campylobacter* possesses only one major porin, that is, MOMP (14). This porin protein forms a trimeric structure on the *Campylobacter* outer membrane, which contains a calcium ion (Ca²^+^) embedded within the constriction zone of the pore, where the Ca²^+^ ion significantly influences the function of MOMP, including its permeability to antibiotics (14). Additionally, mutations in MOMP may influence pathogenesis and virulent phenotypes in *Campylobacter,* as a previous study identified that loop 4 mutations in MOMP were responsible for hypervirulence, which resulted in systemic invasion and abortion in pregnant animals (16).

Some studies further proposed that the expression of β-lactamase OXA-61, a non-carbapenemase, might be associated with carbapenem resistance in *Campylobacter* (7, 9). This class D β-lactamase is encoded by a chromosomal gene *bla*_OXA-61_ and is highly prevalent within this genus (17). The wild-type *bla*_OXA-61_ is characterized by low basal expression and rarely confers antibiotic resistance (17, 18). The *bla*_OXA-61_ gene sequence is highly conserved across genetically diverse strains, typically varying by no more than two nucleotides (17, 18). A single-nucleotide substitution (G → T transversion) in the promoter region of *bla*_OXA-61_ can lead to high-level expression, promoting resistance to several β-lactam antibiotics, though not typically to carbapenems (18). Although the G → T transversion is widespread among *Campylobacter* isolates from diverse sources, only a very small proportion exhibit a carbapenem-resistant phenotype (18, 19). For example, animal-derived *Campylobacter* strains with such an expression-enhanced *bla*_OXA-61_ are quite common (17), but carbapenem resistance has never been reported in these isolates, to our best knowledge. On the other hand, all currently identified strains exhibiting a carbapenem-resistant phenotype are considered to be closely associated with this G → T transversion (5, 7–9). Thus, the association between OXA-61 and carbapenem resistance in *Campylobacter* is still uncertain. Together, these previous findings indicate that the specific mechanism(s) responsible for carbapenem resistance in *Campylobacter* remain unknown. They also suggest the possibility that carbapenem resistance in *Campylobacter* may involve multiple genetic determinants.

Given the critical importance of carbapenems in clinical therapy, it is necessary to understand how *Campylobacter* develops resistance to this class of antibiotics to facilitate effective diagnosis, surveillance, and mitigation. To achieve this goal, we sought to analyze *Campylobacter* evolution under selection pressure from meropenem exposure. From the evolutionary experiment, we identified that mutations associated with two genes, *bla*_OXA-61_ and *porA*, were likely responsible for carbapenem resistance in *Campylobacter*. Subsequent genetic transformation and site-specific mutagenesis confirmed the synergistic role of a *porA* mutation and a *bla*_OXA-61_ promoter mutation in conferring resistance to carbapenems. Structural modeling indicated that the mutation in MOMP resulted in the substitution of a Ca²^+^ coordinating residue (D157H), which altered the conformation and characteristics of the pore, reducing meropenem permeability. Although overexpression of OXA-61, resulting from the promoter mutation, did not exhibit detectable meropenem hydrolytic activity, molecular dynamics (MD) simulations and binding free energy calculations revealed direct binding of meropenem to the drug-binding pocket of OXA-61, which was further validated by the alanine substitution method. Together, our findings convincingly demonstrate that the carbapenem-resistant phenotype in *Campylobacter* is mediated by a synergistic action of mutations in both MOMP and the promoter of *bla*_OXA-61_, which explains the carbapenem resistance phenotype of *Campylobacter* observed in clinical settings.

## RESULTS

### Laboratory evolution of antibiotic-resistant strains

**Fig 1:**
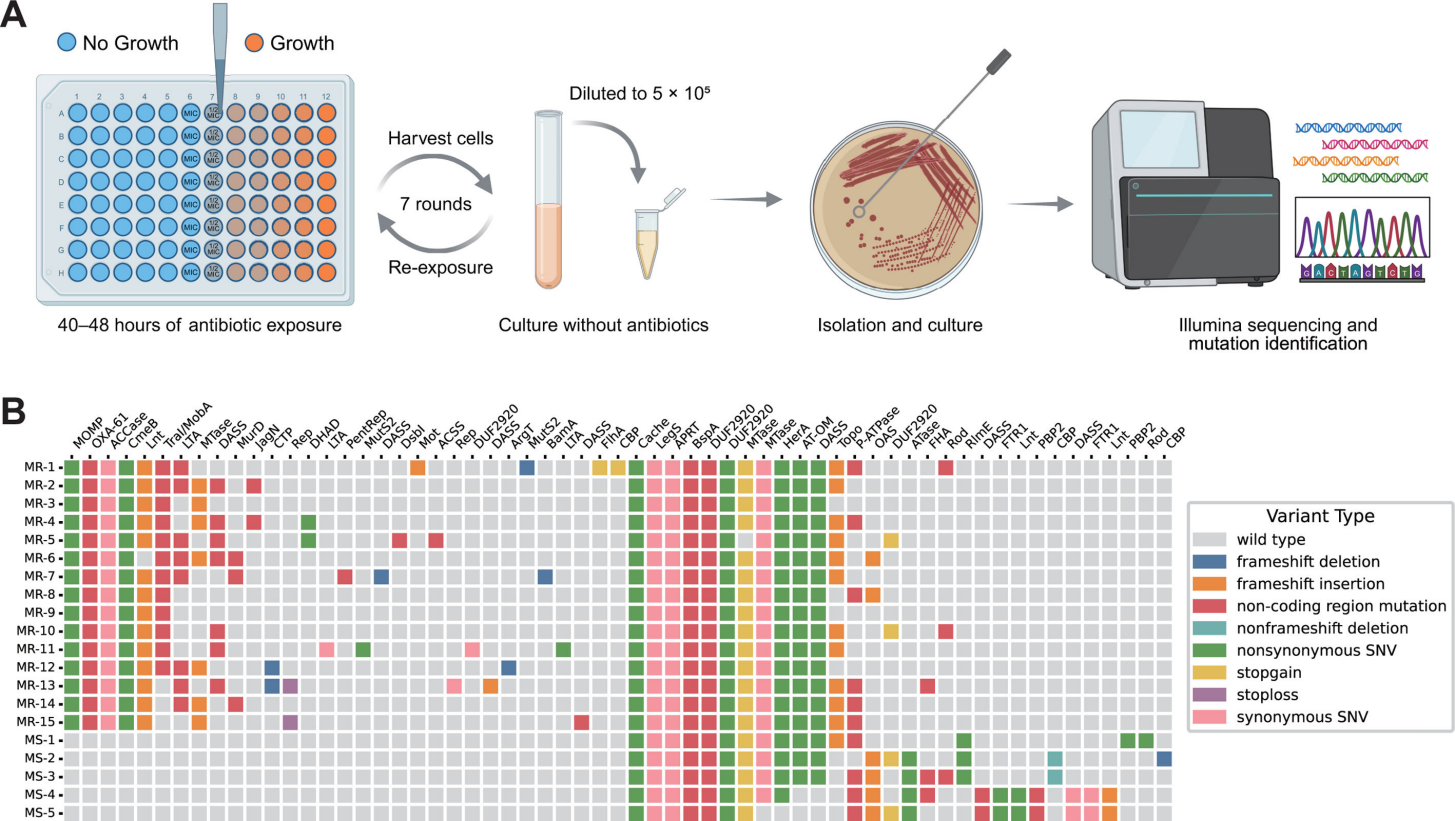
Experimental evolution of carbapenem resistance and comparative genomics-based screening of candidate mutations associated with meropenem resistance. (**A**) Flowchart of the carbapenem resistance evolution experiment. The process involved cyclic antibiotic exposure, antibiotic-free cultivation, monoclonal isolation, and whole-genome sequencing (WGS). (**B**) Identification of putative genetic determinants of meropenem resistance using comparative genomics. A total of 61 mutations from 20 evolved *Campylobacter* strains are shown. Multiple single-nucleotide variants (SNVs) may occur within the same mutated gene. Variants of the same type are merged, while those of different types are listed separately. The heatmap highlights mutated genes that co-occur in MR strains and are absent from MS (meropenem-susceptible) strains (labels on the left).

To decipher how *Campylobacter* develops resistance to carbapenems, we conducted an experimental evolution under antibiotic pressure (Fig. 1A). Following antibiotic exposure and screening, strains derived from ATCC 33559 that were either resistant or remained sensitive to meropenem were obtained. Minimum inhibitory concentration (MIC) measurements revealed that the meropenem-resistant (MR) group exhibited a 16-fold increase (from 0.0625 to 1 μg/mL) in meropenem MIC. A representative isolate, MR-1, is shown in Table 1. In addition, the meropenem-resistant mutants also showed a similar change in the MIC of ampicillin (8-fold) (Table 1). The results demonstrate that *C. coli* ATCC 33559 can rapidly develop resistance to carbapenem under selection pressure.

**TABLE 1:** Meropenem and ampicillin MICs in mutant constructs (μg/mL)

Strain	Meropenem		Ampicillin	
	MIC	Fold change	MIC	Fold change
ATCC 33559	0.0625	1	8	1
MR-1	1	16	64	8
33559 eOXA	0.0625	1	64	8
33559 crMOMP	0.125	2	8	1
33559 eOXA_crMOMP	1	16	64	8
33559 crMOMP/pRY-wOXA	0.125	2	8	1
33559 crMOMP/pRY-eOXA	8	128	≥256	≥32
33559 eOXA/pRY-wOXA	0.125	2	≥256	≥32
33559 eOXA/pRY-eOXA	0.5	8	≥256	≥32
MR-1/pRY-eOXA	8	128	≥256	≥32
33559 ΔOXA	0.0313	0.5	4	0.5
MR-1 ΔOXA	0.125	2	4	0.5
33559 ΔOXA/pRY112	0.0313	0.5	4	0.5
33559 ΔOXA/pRY-S42A-OXA	0.0313	0.5	4	0.5
33559 ΔOXA/pRY-S91A-OXA	0.0313	0.5	8	1
33559 ΔOXA/pRY-eOXA	0.125	2	≥256	≥32

### WGS and comparative genomics analysis identify mutations associated with meropenem resistance

To identify genetic mutations associated with meropenem resistance, we performed WGS on 15 MR *C. coli* isolates (designated MR-1 to MR-15) and five meropenem-sensitive (MS) (designated MS-1 to MS-5) isolates, all derived from the evolutionary experiments. Mutation profiles were constructed and annotated using *C. coli* ATCC 33559 as the reference genome. In the MR strains, we identified 44 mutations in coding regions and 27 in non-coding regions, while the MS strains carried 33 and 20 mutations in coding and non-coding regions, respectively (Fig. 1B). We further screened for mutations present in all MR strains and excluded those also found in MS strains. This comparative analysis identified four candidate genes potentially associated with meropenem resistance: *porA* (encoding the major outer membrane protein, MOMP), *bla*_OXA-61_ (encoding class D β-lactamase OXA-61), *accA* (encoding acetyl-CoA carboxylase), and *cmeB* (encoding the inner membrane transporter of an RND-type efflux system named *cmeABC*) (Fig. 1B).

The mutation in *accA* is a synonymous mutation and was therefore excluded in our initial analysis. The *cmeB* gene is part of *cmeABC*, an efflux system that spans the inner and outer membranes and extrudes various antimicrobial compounds (20). Previous studies using gene knockouts found that *CmeABC* did not affect the susceptibility of *Campylobacter* to carbapenems (21). Additionally, the single point mutation (N294K) was not involved in CmeB binding to antibiotics (22), and natural transformation using the mutated *cmeB* sequence failed to generate meropenem-resistant mutants (data not shown). For these reasons, the *cmeB* mutation was also excluded from further investigation on carbapenem resistance.

Mutations in the *porA* and *bla*_OXA-61_ genes were identified in all of the resistant strains and represented strong candidates for mediating carbapenem resistance. A G → T substitution was identified in the promoter region of the *bla*_OXA-61_ gene, which was previously reported to upregulate its expression (18). An aspartic acid residue was replaced by a histidine in the MOMP of meropenem-resistant isolates. Following the nomenclature for MOMP proposed by Luana et al. (14), we designated the first residue after the signal peptide, Thr1, as the starting point in our numbering system. Within this framework, the mutation was identified as D157H in MOMP. Notably, the same mutation has been reported in a carbapenem-resistant *C. coli* strain isolated from a patient with hematological malignancy (8). Importantly, both the *bla*_OXA-61_ and *porA* mutations have been identified in clinical isolates (7), suggesting their possible role in facilitating *Campylobacter* adaptation under natural conditions.

### Functional characterization of mutations associated with *porA* and *bla*_OXA-61_

To verify the contributions of mutations in *porA* and *bla*_OXA-61_ to carbapenem resistance, site-directed mutagenesis, gene knockout, and plasmid-mediated overexpression were performed in *C. coli* ATCC 33559 and its isogenic mutant strains (Fig. S1). The expression of *bla*_OXA-61_ in all engineered strains was confirmed by the RT-qPCR method (Fig. S2).

For the *porA* gene, it was not possible to construct a knockout mutant as the gene is essential for *Campylobacter* viability (14). However, the D157H mutation was successfully generated by site-directed mutagenesis, and the resultant mutant was designated 33559 crMOMP (Fig. S1). Compared to ATCC 33559 (Table 1), the 33559 crMOMP strain exhibited a twofold increase in the meropenem MIC (0.125 µg/mL). In contrast, ampicillin MIC did not differ from that of the wild-type control (8 µg/mL). This indicates that the D157H mutation alone results in a modest increase in the resistance to carbapenems in *Campylobacter*. For the *bla*_OXA-61_ gene, site-directed mutagenesis was used to generate 33559 eOXA, which harbors the G → T mutation in the promoter, leading to overexpression of the gene (Fig. S1). A deletion mutant of *bla*_OXA-61_ was made to generate 33559 ΔOXA, and plasmid-carried *bla*_OXA-61_ was cloned into 33559 eOXA to increase the copy numbers and expression levels of *bla*_OXA-61_ (33559 eOXA/pRY-wOXA and 33559 eOXA/pRY-eOXA). Above all, four distinct strains with varying levels of OXA-61 expression were obtained by knockout, introduction of the G → T mutation, and introduction of additional *bla*_OXA-61_ copies (33559 eOXA, 33559 ΔOXA, 33559 eOXA/pRY-wOXA, and 33559 eOXA/pRY-eOXA) (Fig. 1C). Both *bla*_OXA-61_-deficient strains and isolates carrying multiple copies of *bla*_OXA-61_ or *bla*_OXA-61_-like genes were found in clinical settings (9, 18, 19, 23). Thus, deletion and plasmid cloning of blaOXA-61 were used to model the expression levels of the gene in various clinical isolates. As determined by RT-qPCR and compared to wild-type ATCC 33559, expression of *bla*_OXA-61_ increased 16-, 34-, and 93-fold in 33559 eOXA, 33559 eOXA/pRY-wOXA, and 33559 eOXA/pRY-eOXA, respectively (Fig. S2). No gene expression was detected in the 33559 ∆OXA strain due to the deletion of the gene. These confirm that the promoter mutation and increased plasmid copy number indeed elevated the expression of the *bla*_OXA-61_ gene. When analyzed for meropenem susceptibility (Table 1), the 33559 eOXA strain did not exhibit increased resistance to meropenem (0.0625 µg/mL), but showed an 8-fold increase in resistance to ampicillin (64 µg/mL) compared to wild-type ATCC 33559. On the other hand, deletion of *bla*_OXA-61_ (33559 ΔOXA) resulted in a twofold decrease in the MICs of meropenem (0.0313 µg/mL) and ampicillin (4 µg/mL). However, overexpression of *bla*_OXA-61_, as shown in the 33559 eOXA/pRY-wOXA strain (0.125 µg/mL) and the 33559 eOXA/pRY-eOXA strain (0.5 µg/mL), led to 2- and 8-fold increases in the resistance to meropenem, respectively, and more than 32-fold increase in the resistance to ampicillin (≥256 µg/mL) (Table 1). These results indicate that *bla*_OXA-61_ contributes to carbapenem resistance in *Campylobacter*, but this contribution is dependent on its expression levels, not just merely the presence of the gene. To determine whether the mutations in *porA* and the expression level of *bla*_OXA-61_ have a synergistic role in carbapenem resistance, we introduced *bla*_OXA-61_ with varying expression levels into the strains with the *porA* mutation background (Fig. S1). Strain 33559 eOXA_crMOMP, which harbored both the G → T mutation in the *bla*_OXA-61_ promoter and the D157H substitution in MOMP, showed 16- and 8-fold increases in the MICs of meropenem (1 µg/mL) and ampicillin (64 µg/mL), respectively, compared to the ATCC 33559 strain (Table 1). These MIC values were identical to those of MR-1, a meropenem-resistant mutant selected from the evolution experiment, and substantially higher than the MICs of either single mutant alone (D157H substitution in MOMP or G → T mutation in the *bla*_OXA-61_ promoter). This result fully explains the phenotype of the MR-1 strain and indicates a significant synergism of the two mutations in mediating meropenem resistance. The importance of synergism was further shown by a 128-fold increase in the meropenem MIC in the MR-1/pRY-eOXA strain when compared to ATCC 33559 (Table 1). Additionally, 33559 crMOMP/pRY-wOXA, which showed a ~8-fold increase in *bla*_OXA-61_ expression compared to the ATCC 33559 strain (Fig. S2), had the same meropenem MIC (0.125 µg/mL) as the 33559 crMOMP strain. However, the 33559 crMOMP/pRY-eOXA strain, in which expression of *bla*_OXA-61_ was increased ~82-fold (Fig. S2), had a meropenem MIC of 8 µg/mL, which is 64- and 16-fold higher than the MIC in the 33559 crMOMP and 33559 eOXA/pRY-eOXA strains, respectively. These results clearly indicate that the synergism between MOMP and *bla*_OXA-61_ in mediating carbapenem resistance is further enhanced by the expression level of *bla*_OXA-61_.

To assess whether this synergistic effect extends to other carbapenems, we evaluated the resistance of the above strains (including 33559 eOXA, 33559 crMOMP, 33559 eOXA_crMOMP, and MR-1) to imipenem and ertapenem. The results showed that neither the single nor the double mutations altered the susceptibility to imipenem (Table 2). For ertapenem, the D157H mutation in MOMP alone led to a 16-fold increase in the MIC (4 µg/mL). However, the G → T transversion alone had no detectable effect on the MIC of ertapenem (Table 2). The combination of the two mutations did not further increase the antibiotic MIC over the effect imposed by the MOMP mutation. These results indicate that the MOMP mutation plays a major role in ertapenem resistance, while overexpression of *bla*_OXA-61_ plays little role in the resistance to the antibiotic.

**TABLE 2:** MICs of carbapenems of differential molecular weights (μg/mL)

Strain	Imipenem (299.35 g/mol)		Meropenem (383.5 g/mol)		Ertapenem (475.5 g/mol)	
	MIC	Fold change	MIC	Fold change	MIC	Fold change
ATCC 33559	0.125	1	0.0625	1	0.25	1
33559 eOXA	0.125	1	0.0625	1	0.25	1
33559 crMOMP	0.125	1	0.125	2	4	16
33559 eOXA_crMOMP	0.125	1	1	16	4	16
MR-1	0.125	1	1	16	4	16

### Conformational changes of the MOMP mutant generated by AlphaFold2

**Fig 2:**
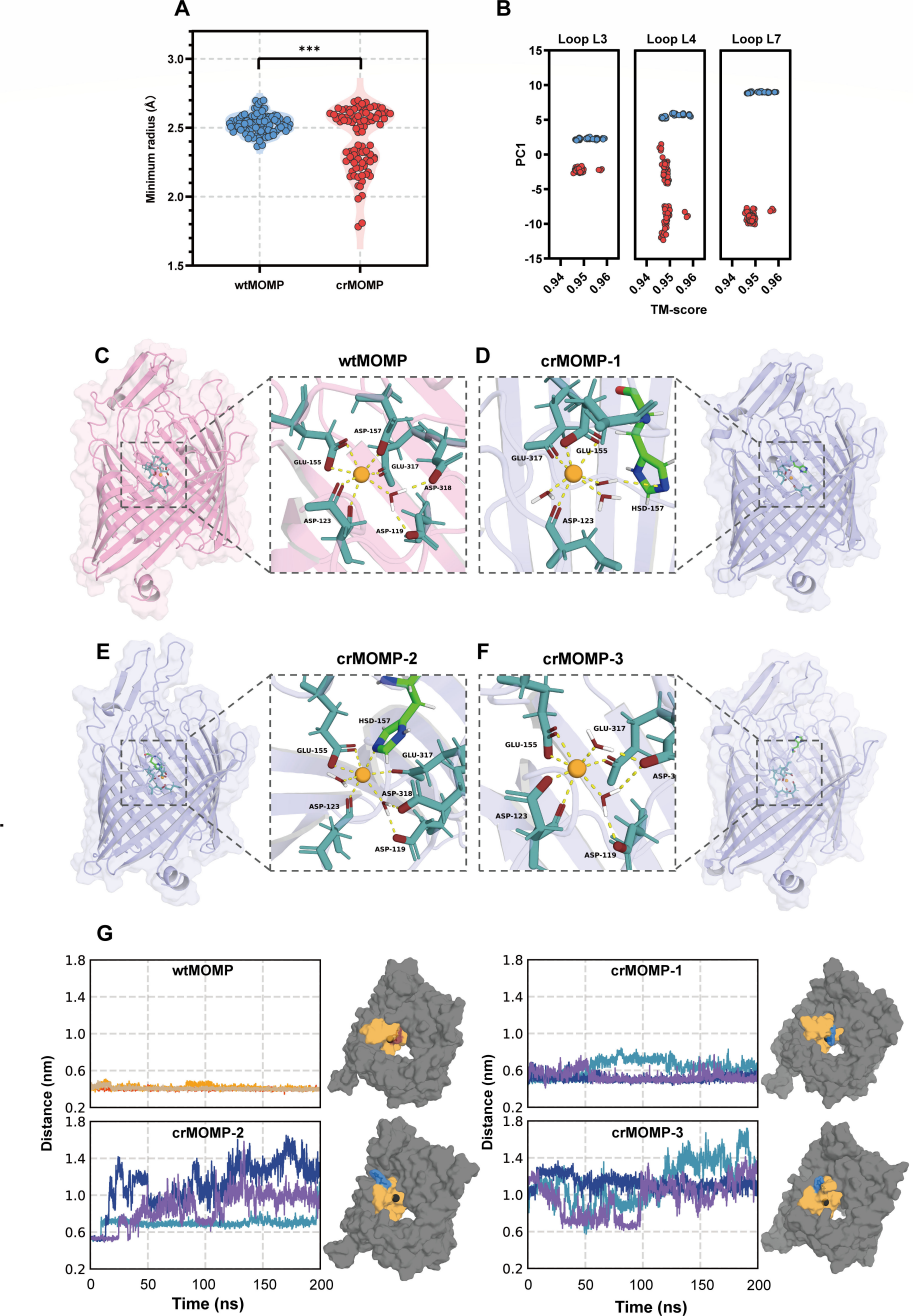
Structural modeling and dynamic characteristics of MOMP. (**A**) Violin plots comparing the minimum channel radii of various MOMP monomers. wtMOMP: wild-type MOMP; crMOMP: carbapenem-resistant MOMP variant. (**B**) Conformational heterogeneity of MOMPs. PCA and TM-scores were used to assess structural variation. The first principal component reflects the distributions of the predicted models, while the TM-score quantifies similarity to the MOMP crystal structure (PDB ID: 5LDT). Blue and red dots represent wtMOMP and crMOMP, respectively. (**C–F**) The Ca^2+^ coordination environment in the MOMP pores. Calcium ions (Ca^2+^) (orange spheres) are coordinated by surrounding amino acids (teal), and residue 157 is highlighted in green. Protein components are shown in cartoon representation, and water molecules are depicted as sticks. (**G**) Dynamics of the Ca^2+^ and residue-157 interaction from MD simulations. The distance between Ca^2+^ and residue 157 was measured over time from three independent MD replicates for each MOMP conformation. The right panel shows a representative channel structure at 200 ns, with extracellular loops (residues 25–44, 73–86, and 159–191) removed for clarity. The Ca^2+^-binding pocket is colored orange. His-157 and Asp-157 are shown in blue and red, respectively. Calcium ions are depicted as black spheres. The distribution of distances between wtMOMP and crMOMP was significantly different (Kolmogorov–Smirnov test, *D* = 0.4333, *P* < 0.001). Statistical significance is indicated with asterisks: *P < 0.05; **P < 0.01; ***P < 0.001; ns, no significant difference.

To understand how the D157H mutation in MOMP may affect carbapenem resistance, we obtained the structures of the wild-type and mutant MOMP using *de novo* structure prediction with AlphaFold2 (24). We modeled 30 trimeric structures for wtMOMP and 30 for crMOMP (60 total). Ramachandran plot analysis confirmed that none of the predicted residues fell into disallowed regions, indicating that the models exhibit good stereochemical quality (Fig. S3) (25, 26). The minimum pore radius of each MOMP monomer was measured using the HOLE2 program (27). The results showed that the average minimum pore radius was quite similar between the 90 (3 × 30) crMOMPs (2.451 Å) and 90 (3 × 30) wtMOMP (2.523 Å) monomers (Fig. 2A). However, crMOMP exhibited greater structural diversity, as reflected in the broader distribution of pore radii (Fig. 2A), suggesting that the D157H mutation substantially influences the minimum pore radius of MOMP. Amino acid residues forming loops L3, L4, and L7 are involved in Ca^2+^ coordination, with residue 157 located in loop L4. Conformational differences of the predicted loops can be delineated using principal component analysis (PCA) and template modeling (TM)-score (Fig. 2B) (28). The results indicated that the D157H mutation led to predicted conformational fluctuations of loop L4. In contrast, the loops (loop 3 and loop 7) without mutation did not exhibit significant conformational variability (Fig. 2B).

Based on the distinct loop L4 conformations identified by the first principal component (PC1), we characterized four representative MOMP conformations (wtMOMP, crMOMP-1, crMOMP-2, and crMOMP-3) (Fig. 2C through F), each exhibiting different Ca^2+^ coordination modes. For wtMOMP, the coordination pattern matched that of the reported MOMP crystal structure (PDB ID: 5LDT) from the *C. jejuni* 85H strain (Fig. S4) (14). Asp-123, Glu-155, Asp-157, and Glu-317, along with a water molecule, formed the Ca^2+^ coordination sphere, with the water further coordinated by Asp-318 and Asp-119 (Fig. 2C; Fig. S4). For crMOMP, the D157H substitution alters the Ca^2+^ coordination in multiple ways. In crMOMP-1, His-157 participates indirectly in Ca^2+^ coordination, with a TIP3P water molecule acting as a mediator (Fig. 2D); in crMOMP-2, His-157 is entirely excluded from the binding pocket (Fig. 2E); and in crMOMP-3, His-157 reoccupies the position originally belonging to Asp-157 and directly coordinates with the Ca^2+^, a coordination mode that is nearly impossible to occur (Fig. 2F). Typically, only aspartic acid and glutamic acid can directly participate in Ca^2+^ coordination (29). Overall, AlphaFold2 predicted multiple conformations of MOMPs. The coordination mode of wtMOMP is well-defined and fully consistent with the experimental structure, whereas crMOMPs exhibit various modes. This modeling result suggests that the D157H mutation is likely deleterious to MOMP, compromising the stability of the constriction zone and impairing drug permeation.

### The multiple effects of the D157H mutation on the pore function of MOMP *in silico*

To further investigate the impact of the D157H mutation on MOMP structure, we performed 200 ns of unbiased MD simulations for both wtMOMP and its variants (crMOMP-1, crMOMP-2, and crMOMP-3) embedded in a lipid bilayer surrounded by a salt solution. We assessed the stability of the Ca^2+^-binding pocket by measuring the root mean square deviation (RMSD) and evaluated the flexibility of loop L4 using root mean square fluctuation (RMSF). The distance between the Ca^2+^ and residue 157 was monitored to track conformational changes within the pocket (Fig. 2G). Distance monitoring indicated that the D157H mutation caused the residue 157 to potentially dissociate from the Ca^2+^-binding pocket. The RMSD profiles indicated that the D157H mutation disrupted pocket stability, consistent with the distance monitoring results (Fig. S5A). The RMSF profile showed that the amino acid substitution resulted in increased flexibility of loop L4 (Fig. S5B). Above all, the MOMP variants showed reduced stability of the Ca^2+^-binding pocket and increased loop L4 flexibility.

Among the three crMOMP models with different initial conformations of loop L4, crMOMP-1 exhibited dynamic characteristics similar to wtMOMP, with residue 157 maintaining a stable distance from the Ca^2+^ in at least two replicates (Fig. 2G; Fig. S5). In the crMOMP-1 initial conformation (Fig. 2D), residue 157 indirectly anchors loop L4, which is involved in Ca²^+^ coordination, thereby still contributing to porin channel stability. These suggest that the position of residue 157 and its coordination status are critical for the stability of the pocket. Given that crMOMP-1 demonstrated relatively stable dynamic characteristics, it was selected as the representative MOMP variant for subsequent analyses and designated as crMOMP.

**Fig 3:**
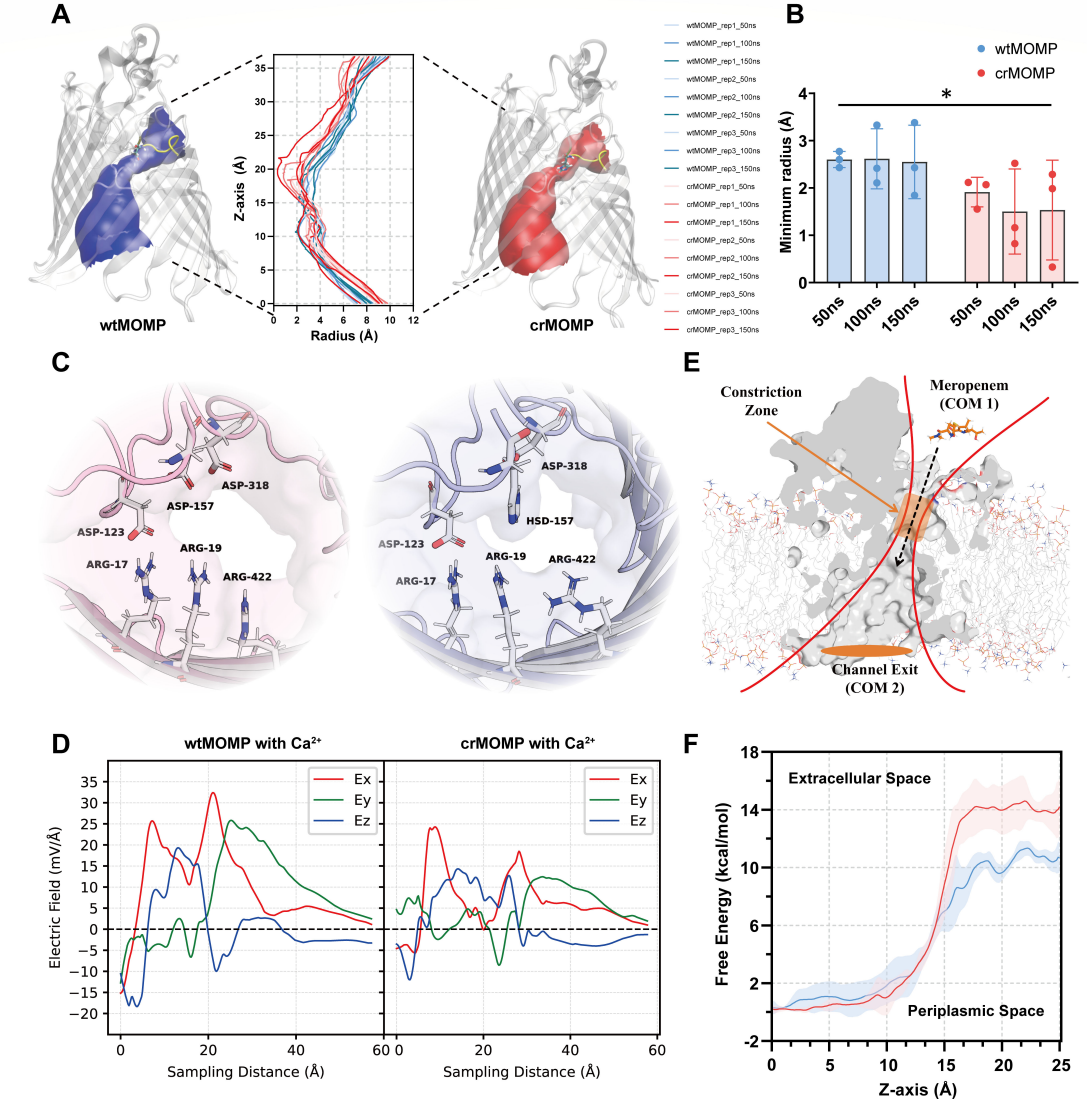
*In silico* modeling of the multiple effects of the D157H mutation on MOMP pore functions. (**A**) Channel radius profiles of wtMOMP and crMOMP at 50 ns, 100 ns, and 150 ns in MD simulations. (**B**) Minimum channel radius of MOMPs in MD. (**C**) Distribution of charged residues within the channel constriction zone. (**D**) Internal electric field along the channel pore. The electric field components along the *X* (red), *Y* (green), and *Z* (blue) axes are plotted relative to the position along the channel centerline. Residue 157 is located at approximately 20–30 Å. (**E**) Solvated meropenem-porin permeation model. The black dashed line indicates the permeation pathway of the drug, and 44 uniformly distributed windows are used to calculate the permeation free energy of the drug. MOMP is displayed as a gray cross-sectional surface, embedded within the layers of POPC phospholipid molecules. Water molecules are not shown. (**F**) The free energy for permeation of meropenem. Mean (solid line) and standard deviation (shaded area) of the free energy for meropenem traversing the constriction zone in both MOMP models. For each system, three independent SMD-REUS simulations were performed to estimate the permeation free energy. The meropenem overcame an additional energy barrier of ~3 kcal/mol to traverse the constriction zone of crMOMP. Significant differences between the wtMOMP and crMOMP groups were analyzed by two-way analysis of variance (ANOVA) followed by Dunnett’s multiple comparisons (*P* < 0.05).

The initial channel radii of wtMOMP and crMOMP were similar, differing by less than 1 Å (Fig. S6). We further estimated the channel radii and minimum channel radii for both MOMPs at 50 ns, 100 ns, and 150 ns during MD simulations (Fig. 3A). The results showed that the channel radius of wtMOMP varied only slightly, with a minimum radius of 1.843 Å. In contrast, crMOMP tended to exhibit a narrower channel radius over the course of the simulations, with a minimum radius of 0.328 Å. On average, the minimum channel radius was significantly larger in wtMOMP (mean: 2.589 Å) than in the crMOMP group (mean: 1.649 Å), with a statistically significant difference between two independent observations (*P* < 0.05) (Fig. 3B). This difference reflects that the constriction zone in the MOMP variants frequently adopts a constricted state, leading to a significant reduction in the minimum channel radii (~1 Å) (Fig. 3B). In contrast, this was not observed in wtMOMP.

The constriction zone of MOMP is formed by a funnel-shaped region with a symmetric arrangement of positive and negative charges, referred to as the eyelet (Fig. 3C; Fig. S7) (14). It is possible that the D157H mutation could partly neutralize the negative charges, thus attenuating the electric field at the eyelet. To test this, we performed electrostatics calculations using APBS and profiled the electric field along the central channel axis (Fig. 3D) (30). The results indicated that crMOMP led to a pronounced reduction in the electric field strength, approximately 30% along the *X*-axis and 40% along the *Y*-axis. The altered electrostatics in MOMP is expected to hinder the translocation of polar molecules.

To evaluate the effect of the D157H mutation on meropenem permeation, we conducted two independent replica exchange umbrella sampling (REUS) simulations for both wtMOMP and crMOMP porin–membrane systems (Fig. 3E). Consistent initial meropenem orientations and permeation pathways were used across all simulations. The initial conformation for each window was derived from steered molecular dynamics (SMD) simulations. The resulting free energy profiles revealed that meropenem faced an additional energy barrier of ~3 kcal/mol when traversing the constriction zone of crMOMP (Fig. 3F; Fig. S8 and S9). This suggested that the D157H mutation impaired porin-mediated meropenem permeation. We propose that both the narrowed constriction zone and the attenuated internal electric field jointly contribute to the permeation barrier, thereby conferring specific carbapenem resistance in *Campylobacter* strains.

### OXA-61 hydrolyzes ampicillin but not meropenem

**Fig 4:**
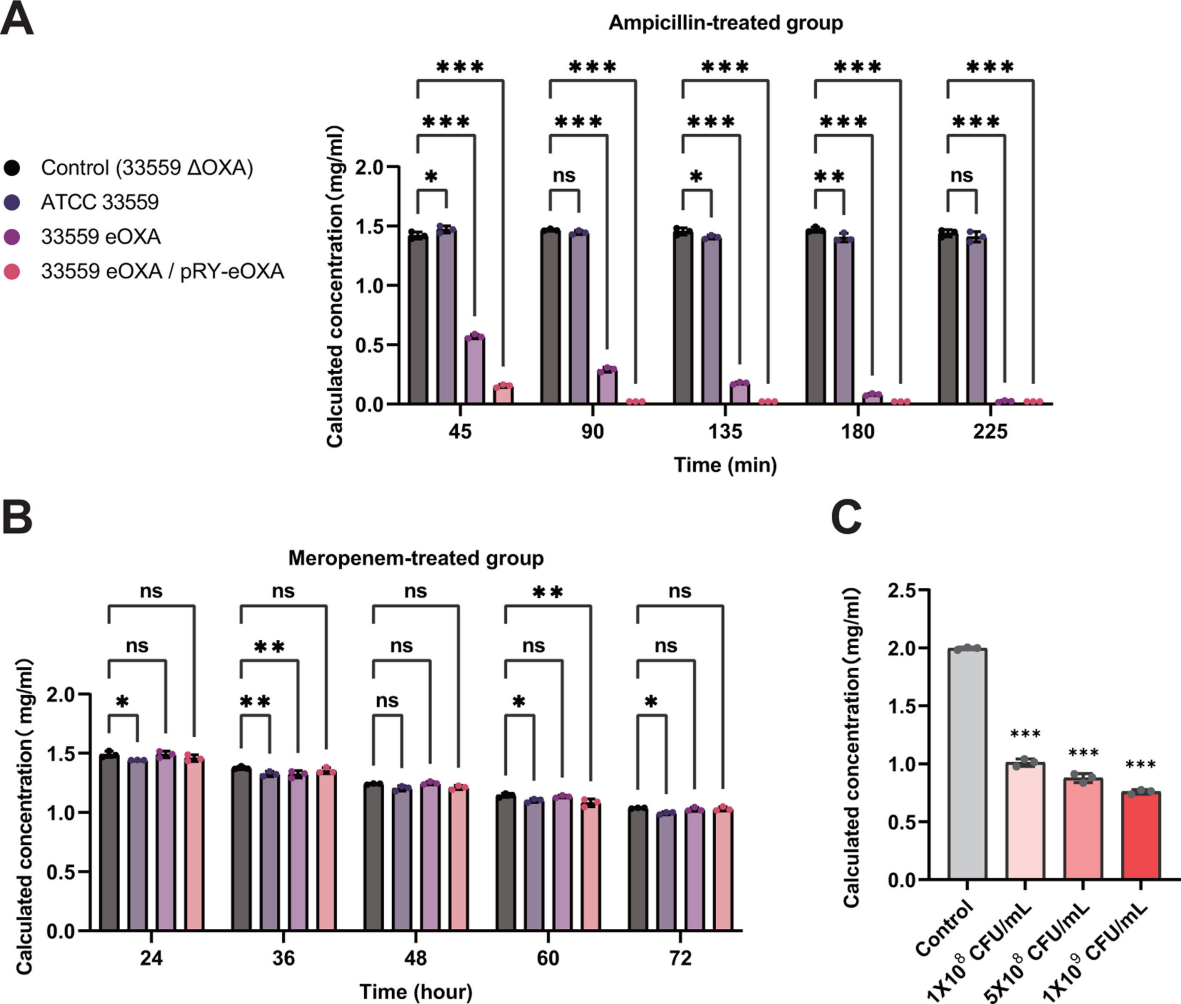
UPLC-based analysis of ampicillin- and carbapenem-hydrolyzing activities. (**A**) Hydrolysis of ampicillin by extracts from *Campylobacter* strains with different *bla*_OXA-61_ expression levels. (**B**) Hydrolysis of meropenem by extracts from *Campylobacter* strains (see the strain denotations in panel A) with different *bla*_OXA-61_ expression levels. The statistical significance was determined by a two-way ANOVA followed by Dunnett’s multiple comparisons. (**C**) Meropenem hydrolysis assay of periplasmic extracts from *E. coli* producing NDM-5. Three different numbers of *E. coli* cells were used in the assay, with the lysis buffer (without bacterial cells) used as the control group. Group differences were analyzed using Welch’s and Brown-Forsythe ANOVA, followed by multiple comparisons against the control group using the Dunnett test. All treatment groups showed statistically significant reductions in meropenem levels compared to the control group (*P* < 0.001 for all comparisons). In all three panels, statistical significance is indicated with asterisks: *P < 0.05; **P < 0.01; ***P < 0.001.

To clarify how OXA-61 expression contributes to meropenem resistance, we compared its hydrolytic activity against ampicillin and meropenem using ultra-high performance liquid chromatography (UPLC). Periplasmic extracts from strains with varying *bla*_OXA-61_ expression levels were incubated with each antibiotic at identical concentrations. The results showed that periplasmic extracts from the strain carrying multiple copies of expression-enhanced *bla*_OXA-61_ (33559 eOXA/pRY-eOXA) efficiently hydrolyzed ampicillin, with over 80% of the drug hydrolyzed within 45 minutes (Fig. 4A). The strain with a single copy of expression-enhanced *bla*_OXA-61_ (33559 eOXA) also demonstrated significant ampicillin hydrolysis activities but required more than 135 minutes to achieve 80% hydrolysis (Fig. 4A). In contrast, extracts from ATCC 33559 and 33559 ∆OXA showed negligible ampicillin degradation. Different from ampicillin, no detectable meropenem hydrolysis by the extract was observed, regardless of the *bla*_OXA-61_ expression level (Fig. 4B). In all cases, neither meropenem hydrolysis products nor the meropenem-derived lactone were detected in any of the OXA-61-expressing strains. As a positive control, periplasmic extracts from an *E. coli* strain SC12 carrying the known carbapenemase gene *bla*_NDM-5_ hydrolyzed meropenem efficiently (Fig. 4C). These findings indicate that OXA-61 does not function as a carbapenemase and suggest that its contribution to carbapenem resistance is not related to a direct hydrolysis of carbapenem.

### *In silico* analysis of OXA-61–drug interactions reveals stable binding of meropenem

**Fig 5:**
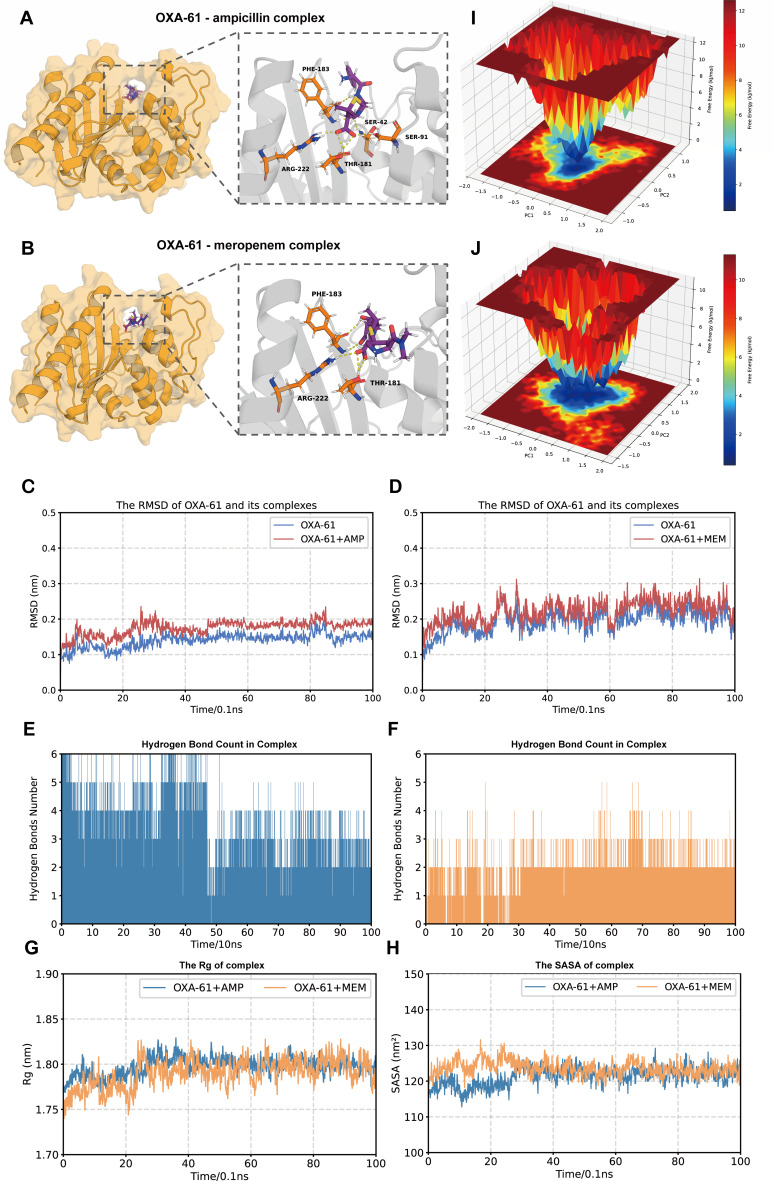
*In silico* analysis of OXA-61 interaction with antibiotics. (**A and B**) Molecular docking poses of OXA-61 with ampicillin (AMP) and meropenem (MEM). OXA-61 is displayed as a yellow cartoon and surface model in the left panel, while interactions between OXA-61 (yellow) and a drug (purple) are depicted using the stick representation in the right panel. (**C and D**) RMSD curves of OXA-61 in complex with ampicillin and meropenem throughout the MD simulation. (**E and F**) Number of hydrogen bonds between OXA-61 and ampicillin (left) and meropenem (right) over the course of MD simulation. (**G**) The Rg curves of OXA-61–drug complexes. (**H**) The SASA curves of OXA-61–drug complexes. (**I and J**) Free energy landscape of OXA-61 complex with ampicillin (top) and meropenem (bottom).

Although OXA-61 lacks a detectable carbapenemase activity, it remains possible that it binds to meropenem and consequently reduces the concentration of free meropenem in the periplasmic space. To investigate this possibility, the β-lactamase OXA-61 structure was obtained via AlphaFold2 with default parameters (31). The protonated ampicillin and meropenem molecules were then individually docked into the drug-binding pocket of OXA-61 (32). In the initial docking configuration, the carbonyl and carboxyl groups of the β-lactam molecules were positioned deep within the binding pocket, enabling the formation of hydrogen bonds and salt bridges (Fig. 5A and B). The OXA-61–drug complexes were subsequently subjected to 100 ns MD simulations. RMSD analysis revealed that both complexes attained equilibrium after 20 ns of simulation, with fluctuations around 0.2 nm, which is within the acceptable range for natural protein dynamics (Fig. 5C and D). Hydrogen bond analysis confirmed the presence of 2–4 hydrogen bonds in both complex systems (Fig. 5E and F). Radius of gyration (Rg) analysis revealed similar levels of structural compaction and stable dynamics for both complexes throughout the simulation (Fig. 5G). Solvent accessible surface area (SASA) analysis revealed that both systems exhibited consistent and stable solvent exposure (Fig. 5H). Free energy landscape (FEL) analysis based on PCA demonstrated that OXA-61 in both complexes occupied a single dominant minimum energy well, indicating no major conformational changes (Fig. 5I and J). From the 2D ligand–protein interaction diagrams, key residues and functional groups involved in the binding interaction were identified (Fig. S10). For ampicillin, the carboxyl group of the thiazolidine ring and the carbonyl group of the β-lactam ring were critical for interactions with OXA-61, primarily via hydrogen bonding and salt bridges (Fig. S10). Similar interaction sites and modes were observed for meropenem. Collectively, the MD analysis suggests that stable binding interactions between OXA-61 and meropenem are feasible.

Furthermore, the binding affinities of the drug–OXA-61 complexes were evaluated using the well-established MM/PBSA method (33–35). The average binding free energy calculated for ampicillin (−34.38 ± 0.08 kcal/mol) and meropenem (−29.15 ± 0.18 kcal/mol) indicates that both compounds are able to establish strong interactions with OXA-61 (Table 3). Electrostatic (Δ*G*_ele_) and Van der Waals (Δ*G*_vdw_) interactions were identified as key contributors to stabilizing drug binding (Table 3). It is evidenced that the binding between meropenem and OXA-61 is thermodynamically spontaneous.

**TABLE 3:** Binding free energy decomposition (kcal/mol)^*a*^

Complex	Δ*G*_vdw_	Δ*G*_ele_	Δ*G*_MM_	Δ*G*_polar_	Δ*G*_apola*r*_	Δ*G*_sol_	Δ*G*
Ampicillin + OXA-61	−29.73 ± 0.10	−148.43 ± 0.65	−178.16 ± 0.63	147.64 ± 0.54	−3.86 ± 0.00	143.78 ± 0.65	−34.38 ± 0.18
Meropenem + OXA-61	−22.93 ± 0.09	−52.85 ± 0.28	−75.78 ± 0.28	50.2 ± 0.22	−3.57 ± 0.00	46.63 ± 0.22	−29.15 ± 0.18

^*a*^ Δ*G*_vdw_: Van der Waals energy; Δ*G*_ele_: electrostatic energy; Δ*G*_MM_: molecular mechanics free energy; Δ*G*_polar_: polar solvation free energy; Δ*G*_apolar_: non-polar solvation free energy; Δ*G*_sol_: solvation free energy.

### Alanine substitutions in OXA-61 eliminate meropenem resistance

Binding free energy decomposition revealed that two serine residues (SER-42 and SER-91) located within the drug-binding pocket participated in binding interactions and contributed substantially to the binding energy (Fig. S11). The SER-42 is located within the STFK active-site tetrad and participates in the addition–elimination reaction carried out by the nucleophilic serine residue with a substrate β-lactam (36). The SER-91 is located in another motif of OXA-61, a conserved amino acid sequence near the active site that is generally considered to be involved in the correct orientation of the substrate (37). Both serine residues are deeply buried at the bottom of the drug-binding pocket (Fig. 5A and B). To examine the functional roles of these two residues, we performed alanine substitutions. The corresponding *bla*_OXA-61_ gene variants were individually cloned into the pRY112 plasmid and introduced into the strain 33559 ΔOXA (Fig. S1). Two alanine-substituted mutants of OXA were generated (33559 ΔOXA/pRY-S42A-OXA and 33559 ΔOXA/pRY-S91A-OXA). Both variants carried a common promoter mutation (G → T), enabling high-level expression. The 33559 ΔOXA/pRY112 strain and 33559 ΔOXA/pRY-eOXA strain served as controls. Their construction is presented in Fig. S1. The expression of *bla*_OXA-61_ was confirmed by RT-qPCR (Fig. S12). The antimicrobial susceptibility testing showed that either alanine substitution abolished resistance to meropenem and ampicillin (Table 1). Given that the residues are engaged in specific binding to meropenem in the drug-binding pocket of OXA-61, the MIC results suggest that meropenem binding directly contributes to the resistance. Notably, substitution of the non-catalytic SER-91 also completely eliminated meropenem resistance, suggesting that non-covalent interactions are the key determinants of the binding process. Collectively, these data indicate that meropenem–OXA-61 binding interaction contributes to the observed resistance and is further modulated by OXA-61 expression levels.

## DISCUSSION

**Fig 6:**
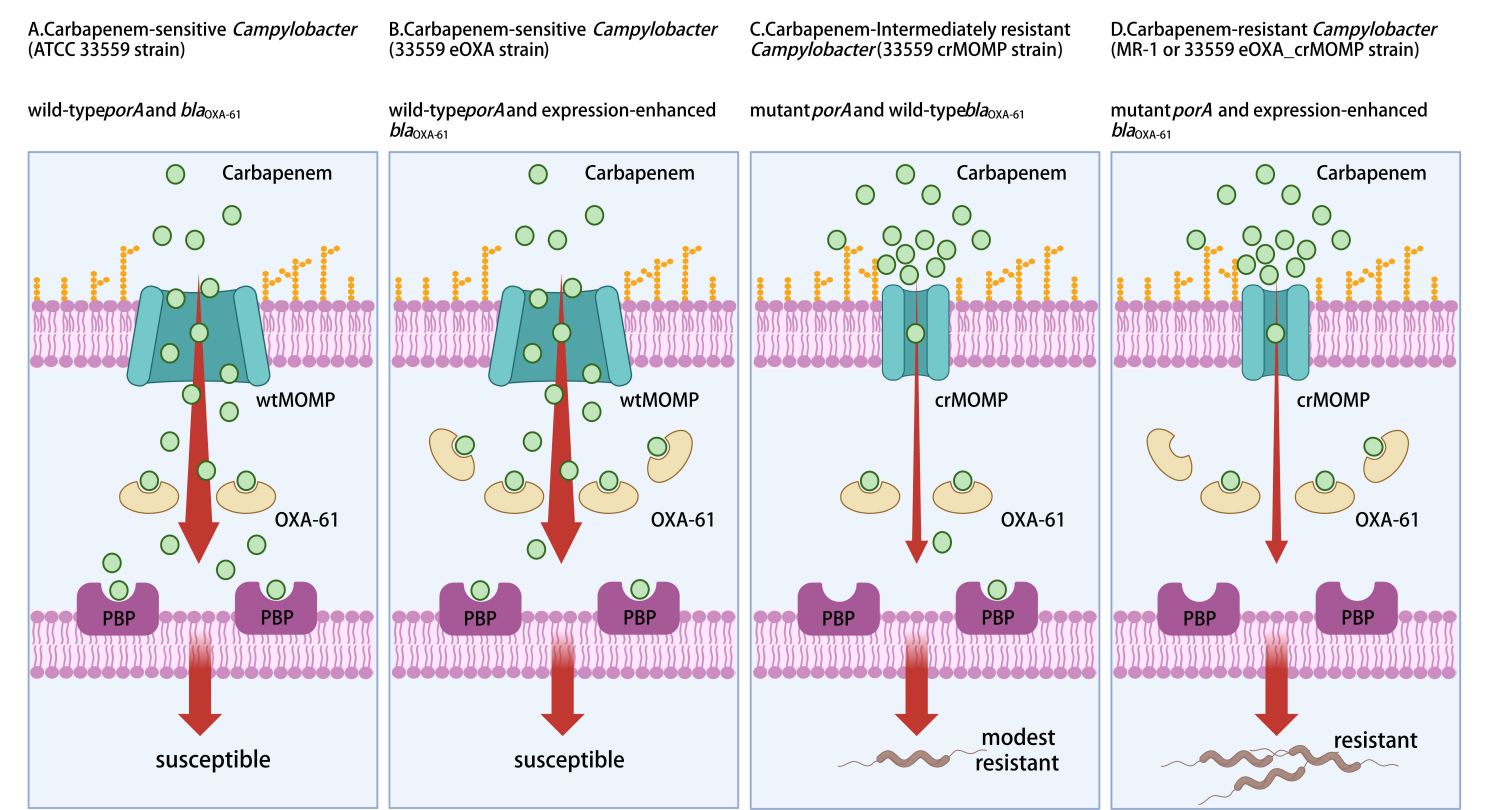
Diagrams illustrating the synergistic mechanism of carbapenem resistance in *Campylobacter*. (**A**) In ATCC 33559 (wild type), the MOMP permits high carbapenem influx, and the basal expression of OXA-61 is far from sufficient to sequester the drug, leading to its binding to and inhibition of penicillin-binding proteins (PBPs). (**B**) In 33559 eOXA (expression-enhanced *bla*_OXA-61_), strong expression of OXA-61 contributes to carbapenem sequestration. However, the high influx overwhelms the sequestration by OXA-61, and PBPs are still inhibited. (**C**) In 33559 crMOMP (reduced influx), the MOMP mutation reduces carbapenem influx. Carbapenems accumulate within the periplasm at a very low rate. Although OXA-61 is not overproduced, the low drug concentration reduces the damage to PBPs. (**D**) In MR-1 or 33559 eOXA_crMOMP (synergistic action), the combination of crMOMP (reducing influx) and enhanced OXA-61 expression (efficiently sequestering the drug within the periplasmic space) works synergistically to prevent carbapenem binding to PBPs and thereby confers carbapenem resistance.

Carbapenem-resistant bacteria, particularly among *Enterobacteriaceae,* have become a major clinical concern (38). Carbapenem is also a critical antibiotic reserved for treating infections caused by multidrug-resistant or extensively drug-resistant *Campylobacter* (39, 40). However, non-carbapenemase-producing but carbapenem-resistant *Campylobacter* isolates have been increasingly identified and reported in clinical settings (5, 7–9), underscoring the need to understand how *Campylobacter* evolves carbapenem resistance. In this research, we report a novel carbapenem resistance mechanism that involves a synergistic action of mutations associated with *porA* and *bla*_OXA-61_. First, mutations in the *porA* gene lead to structural and functional transitions in the encoded porin protein, impeding carbapenem permeability (Fig. 3). Second, sustained and high-level expression of the β-lactamase OXA-61 results in the sequestration of carbapenems within the periplasmic space, thereby reducing the availability of carbapenems to penicillin-binding proteins involved in peptidoglycan synthesis of the bacterial cell wall (Fig. 5). A model illustrating this synergistic mechanism is shown in Fig. 6A through D. Importantly, the two pathways independently can only confer mild resistance to meropenem (2- to 8-fold), while their synergistic cooperation significantly enhances the resistance level (16- to 128-fold) (Table 1). This synergistic mechanism fully explains the carbapenem resistance observed in clinical settings as both *porA* mutations and *bla*_OXA-61_ overexpression have been reported in clinical carbapenem-resistant *Campylobacter* isolates (5, 7–9).

Typically, spontaneous mutation and horizontal gene transfer are the main pathways by which bacteria acquire antibiotic resistance (41). OXA-48-like carbapenemases represent one of the most common types of carbapenemases in *Enterobacterales*. The global dissemination of *bla*_OXA-48_-like genes is mainly attributed to their highly compatible plasmids, such as IncL, IncX3, and ColE2 (11). To date, no studies have demonstrated the presence of carbapenemase- or β-lactamase-encoding plasmids in *Campylobacter*. Both *bla*_OXA_-61-like and *porA* genes are located on the chromosome, whose mutations mediate carbapenem resistance in *Campylobacter*. We first identified the putative carbapenem resistance mutations through laboratory evolution and then verified their specific role using site-specific mutagenesis and plasmid cloning for gene overexpression. Additionally, structural modeling provided further information on the mechanistic aspects of the resistance mechanism. Together, these findings convincingly showed that mutations in *porA* and the promoter of *bla*_OXA-61_ enable rapid development of resistance to carbapenems, providing an efficient strategy for *Campylobacter* to cope with carbapenem selection pressure.

To date, the mechanisms underlying carbapenem resistance in non-carbapenemase-producing bacterial strains remain an emerging area of knowledge (13). In general, carbapenem resistance in non-carbapenemase-producing strains often requires the accumulation of multiple genetic changes (42–44). Reports of such a mechanism are increasing and are mainly associated with mutations or loss of outer membrane proteins (45). For example, strains producing the non-carbapenemase OXA-10 can develop carbapenem resistance when porins are absent (46). In another case, non-carbapenemase-producing *Klebsiella* strains acquired carbapenem resistance through the carriage of a megaplasmid (pNAR1) encoding the β-lactamase DHA-1 and an inactivating mutation in the chromosomal gene *ompK36* (encoding porin ompK36) (13). Extensive clinical reports indicate that the expression of *bla*_ESBL_ and *bla*_AmpC_ can confer carbapenem resistance in *Enterobacterales* characterized by porin mutations or loss. In certain regions, the prevalence of such strains among carbapenem-resistant *Enterobacterales* is strikingly high (42–44). However, in the case of *Campylobacter*, the meropenem resistance-conferring mutation in *porA* did not inactivate the porin. Instead, it shifted the porin into a conformational state characterized by reduced permeability to carbapenems. This could be explained by the fact that *porA* is an essential gene and a complete loss of its function would be lethal to *Campylobacter* (14, 16). In summary, porin loss, inactivating mutations, or internal conformational shifts can all result in reduced permeability to carbapenems, which constitutes one of the key determinants of carbapenem resistance in non-carbapenemase-producing strains.

Highly expressed non-carbapenemase β-lactamase may contribute to carbapenem resistance. It has been reported that the expression of plasmid-encoded *bla*_TEM_, *bla*_SHV_, *bla*_CTX-M_, *bla*_DHA_, and certain *bla*_OXA_ variants can be induced by β-lactams, leading to constitutive high-level expression, which is considered another pivotal characteristic of carbapenem resistance (42). In this study, we showed that carbapenem resistance is correlated with the expression level of *bla*_OXA-61_. In a *porA* mutant genetic background, when the expression-enhanced *bla*_OXA-61_ gene is present in multiple copies, it can confer stronger carbapenem resistance (8 µg/mL) (Table 1), which is consistent with clinical observations (4–32 µg/mL), where *bla*oxa_-61_-like genes, such as *bla*oxa_-61_ and *bla*_OXA-489_, are overexpressed in carbapenem-resistant *Campylobacter* (12). Importantly, our results indicate that OXA-61 contributes to carbapenem resistance by sequestrating the antibiotic (Fig. 4 to 6), instead of hydrolyzing it. This mechanism could be extended to other bacterial species where non-carbapenemase β-lactamases are associated with carbapenem resistance (13, 46). Based on these previous findings and results from this study, we propose that the joint action of reduced membrane permeability and enhanced expression of non-carbapenemase β-lactamases is a general mechanism for non-carbapenemase-mediated resistance to this class of antibiotics (Fig. 6A through D).

A previous study indicated that the G → T substitution in the promoter of *bla*_OXA-61_ dramatically increased the expression of the gene in *C. jejuni* (~239-fold) (18). However, the same substitution in the *C. coli* isolate used in this study only resulted in a ~18-fold increase in *bla*_OXA-61_ expression (Fig. S1). In the same *C. coli* strain carrying multiple copies of *bla*_OXA-61_ with the G → T substitution in the promoter, the expression level increased ~80-fold compared to the wild-type strain (Fig. S1). These differences in the overexpression level between species could be explained by a very low basal level of expression of *bla*_OXA-61_ in *C. jejuni* (18). Cases of carbapenem resistance have been reported in both *C. coli* and *C. jejuni* (5, 7–9). Without exception, *bla*_OXA-61_ with a G → T substitution was identified in all such cases.

To date, reported mutations in the MOMP of carbapenem-resistant *Campylobacter* strains are mainly localized to the L3, L4, and L7 loops, which coordinate with Ca^2+^ (Fig. S13) (5, 7–9). The D157H substitution identified in this study resides in the L4 loop. Notably, L4 loop mutations have been previously associated with increased virulence in animal-derived *Campylobacter* and have been implicated in ovine abortion cases in the United States (16). This implies that certain MOMP mutations may confer both antibiotic resistance and enhanced pathogenicity. Therefore, whether mutations conferring carbapenem resistance affect *Campylobacter* pathogenesis remains to be examined in future studies.

Interestingly, we observed that the synergistic mechanism confers resistance to meropenem and ertapenem but is ineffective against imipenem (Table 2). This finding is consistent with observations made in previous studies, which noticed that meropenem- and ertapenem-resistant *Campylobacter* isolates remained susceptible to imipenem (5, 9). One possible explanation for this variable effect is the difference in molecular weights among the three drugs, with imipenem being the smallest in size (Table 2). We propose that the D157H mutation is particularly effective against larger carbapenems, such as meropenem and ertapenem, by hindering antibiotic penetration. Permeation of large molecules may require overcoming a higher energy barrier through the mutated porin, whereas smaller molecules may be unaffected. Specifically, when the molecular mass of the drug is within a moderate range, as seen with meropenem (Table 2), the D157H mutation reduces the drug permeation across the outer membrane, but its impact remains limited. In this case, antibiotic sequestration regulated by the expression level of *bla*_OXA-61_ plays a more prominent role in the resistance to meropenem. Conversely, for drugs with larger molecular masses, such as ertapenem (Table 2), the effect of the D157H mutation in MOMP plays a major role in the resistance because the mutation prevents ertapenem entry into the periplasmic space. Consequently, overexpression of OXA-61 is no longer needed for ertapenem resistance (Table 2). Thus, the relative contribution of each of the two mutation mechanisms in the synergistic action varies with the sizes of carbapenems. Based on the findings from this study and published by others (5, 9), we hypothesize that the use of small-molecular-weight carbapenems for therapeutic purposes may help mitigate or delay the development of carbapenem resistance in *Campylobacter*. However, whether *Campylobacter* can further develop resistance to imipenem and whether imipenem represents a more effective therapeutic option remains to be determined in future studies. There are two limitations in our study. First, we did not obtain pure β-lactamase OXA-61 via either *E. coli* or yeast expression systems, which precluded further investigation into the precise interactive mode and enzymatic kinetics. Second, the impact of the D157H mutation on MOMP function may have been underestimated, since Ca^2+^ assembly is likely to occur at a very early stage (14). The process of Ca^2+^-porin assembly and the impact of mutations thereon remain unknown. Our interpretation of the mutation effect relies on an assumption that Ca^2+^ can still form a proper coordination pattern with other structural loops. At present, studies on the structure and function of MOMP in *Campylobacter* are limited. Further research into carbapenem resistance mechanisms related to MOMP, particularly through protein structural analysis, is necessary for future advances in this field.

## MATERIALS AND METHODS

### Bacterial strain cultivation

*Campylobacter* strains were cultured on Mueller–Hinton agar (MHA) or in Mueller–Hinton broth (MHB) under microaerophilic conditions (85% N₂, 10% CO₂, 5% O₂) at 42°C for 24–48 hours (47). *E. coli* strains were grown on MHA plates at 37°C. When necessary, antibiotics were supplemented at the following concentrations for transformant selection: kanamycin (30 µg/mL), ampicillin (100 µg/mL), trimethoprim (10 µg/mL), chloramphenicol (30 µg/mL), and meropenem (0.5 µg/mL).

### Antimicrobial susceptibility test

The susceptibility of *C. coli* ATCC 33559 and its derivatives to meropenem, imipenem, ertapenem, and ampicillin was evaluated by broth microdilution. Minimum inhibitory concentrations were determined and interpreted following the European Committee on Antimicrobial Susceptibility Testing (EUCAST) guidelines. It should be noted that neither EUCAST nor the Clinical and Laboratory Standards Institute (CLSI) has established clinical breakpoints for carbapenem resistance in *Campylobacter. C. coli* ATCC 33559 was included as a quality control strain.

### Antibiotic resistance evolution experiment

*C. coli* ATCC 33559 was used as the parental strain for experimental evolution of carbapenem resistance. The strain was initially cultured in antibiotic-free MHB for 24 hours and adjusted to approximately 1 × 10^6^ CFU/mL. This standardized suspension was used to inoculate 96-well plates containing twofold serial dilutions of meropenem (0.0625–4 μg/mL). After incubation for 40–48 hours, bacteria from the well with the highest meropenem concentration that still supported growth were collected and subcultured in antibiotic-free MHB for a 24-hour recovery phase. The bacterial suspension was then standardized again (1 × 10^6^ CFU/mL), and the cycle of reinoculation and antibiotic exposure using 96-well plates was repeated. Following seven rounds of selection, the final resuscitated bacterial suspension was collected and stored. The meropenem resistance evolution experiment was performed in three independent parallel replicates. Each evolved population was streaked onto agar plates, and after incubation, single colonies were selected. To assess phenotypic stability, every single colony underwent 10 rounds of serial passage in antibiotic-free medium. Isolates that maintained stable MICs were preserved and subjected to whole-genome sequencing by the Illumina platform (Sangon Biotech).

### Whole-genome sequencing and comparative genomics analysis

WGS was performed using the Illumina platform on 20 *C. coli* isolates obtained from the meropenem resistance evolution experiment, including 15 meropenem-resistant mutants and five meropenem-sensitive mutants. Genomic assembly was performed with the SPAdes Assembly Toolkit (48), followed by processing of the high-throughput sequencing data with Samtools to extract genomic variant features (49). For functional annotation, a customized *C. coli* genome annotation database was constructed with ANNOVAR, based on GTF files from the NCBI assembly (accession: GCF_900446355.1) (50). Comparative genomic analysis was then carried out to identify and annotate SNVs and insertions/deletions (InDels). Additionally, WGS was applied to a series of genetic mutants constructed in this study, to confirm the intended mutation sites and to screen for any potential off-target events.

### Construction of *C. coli* mutants for functional analysis of carbapenem resistance

To investigate the roles of specific mutations in mediating carbapenem resistance, a series of isogenic mutants was generated from the parental strain *C. coli* ATCC 33559 via targeted genetic manipulation. The suicide plasmid pGEM-T Easy (51) served as the backbone for constructing gene-replacement vectors. Each vector contained dual homology arms, a kanamycin resistance (Kanᴿ) cassette, and a mutated allele of either the *porA* or *bla*_OXA-61_ gene. These constructs designated pGEM-OXA-KAN and pGEM-MOMP-KAN were cloned into *E. coli* DH5α, followed by plasmid purification and quantification using a Thermo Scientific NanoDrop 2000.

For transformation, 1–2 µg of the respective plasmid (pGEM-OXA-KAN and pGEM-MOMP-KAN) was introduced into *C. coli* ATCC 33559 via electroporation. Cells were immediately recovered in 900 µL of MHB and incubated under microaerophilic conditions at 42°C for 6–12 hours. The culture was then centrifuged, and the pellet was resuspended in 200 µL of MHB before plating on Mueller-Hinton agar (MHA) supplemented with 30 µg/mL kanamycin. After 2–4 days of incubation, candidate transformants were selected. Correct integration was verified by PCR and Sanger sequencing. Each verified mutant was subsequently subjected to WGS to confirm the presence of the intended mutation and the absence of unintended genetic changes. The resultant strains were designated 33559 eOXA (promoter mutant of *bla*_OXA-61_) and 33559 crMOMP (*porA* mutant), respectively.

A double mutant harboring both mutations was constructed by electroporating the *bla*_OXA-61_ mutant plasmid (pGEM-OXA-KAN) into the 33559 crMOMP strain. Transformants were selected on MHA plates containing chloramphenicol and confirmed by sequencing. This double mutant was designated 33559 eOXA_crMOMP. Knockout strains for *bla*_OXA-61_ (33559 ΔOXA and MR-1 ΔOXA) were constructed analogously. The knockout plasmid pGEM-KO-OXA-KAN was generated by cloning a Kanᴿ cassette, flanked by ~ 1.0 kb homologous sequences upstream and downstream of the *bla*_OXA-61_ gene, into the pGEM-T Easy vector backbone.

To modulate *bla*_OXA-61_ expression levels, engineered shuttle plasmids were introduced into various genetic backgrounds. The plasmids pRY-wOXA and pRY-eOXA were constructed using pRY112 (52) as the backbone. pRY-wOXA carries the wild-type *bla*_OXA-61_ gene and its native promoter, while pRY-eOXA carries the gene with a G → T transversion in its promoter, leading to enhanced expression. These plasmids were electroporated into recipient strains (e.g., 33559 crMOMP, 33559 ΔOXA, or MR-1), and transformants were selected on MHA containing 30 µg/mL chloramphenicol, generating a panel of strains for assessing gene expression in different genetic contexts. These bacterial strains are listed in Table 4.

**TABLE 4:** Bacterial strains used in this study

Strain	Genotype^*a*^	Phenotype^*b*^	Source
ATCC 33559	—^*c*^	Mem^s^	ATCC
MR-1	*bla*_OXA-61_ promoter(−57G>T) *porA*^G535C	Mem^r^, Amp^r^	This study
33559 eOXA	*bla*_OXA-61_ promoter(−57G>T)	Amp^r^	This study
33559 crMOMP	*porA*^G535C *porA*(3')::kan	Mem^r^, Kan^r^	This study
33559 eOXA_crMOMP	*porA*^G535C *porA*(3')::kan *bla*_OXA-61_ promoter(−57G>T)	Mem^r^, Amp^r^, Kan^r^	This study
33559 ΔOXA	*bla*_OXA-61_::kan	Kan^r^	This study
MR-1 ΔOXA	*bla*_OXA-61_::kan	Mem^r^, Kan^r^	This study
33559 crMOMP/pRY-wOXA	*porA*^G535C *porA*(3')::kan/pRY-wOXA	Mem^r^, Kanr, Cm^r^	This study
33559 crMOMP/pRY-eOXA	*porA*^G535C *porA*(3')::kan/pRY-eOXA	Mem^r^, Amp^r^, Kan^r^, Cm^r^	This study
33559 eOXA/pRY-wOXA	*bla*_OXA-61_ promoter(−57G>T)/pRY-wOXA	Amp^r^, Cm^r^	This study
33559 eOXA/pRY-eOXA	*bla*_OXA-61_ promoter(−57G>T)/pRY-eOXA	Mem^r^, Amp^r^, Cm^r^	This study
MR-1/pRY-eOXA	*bla*_OXA-61_ promoter(−57G > T) *porA*^G535C/pRY-eOXA	Mem^r^, Amp^r^, Cm^r^	This study
33559 ΔOXA/pRY-S42A-OXA	*bla*_OXA-61_::kan/pRY-S42A-OXA	Kan^r^, Cm^r^	This study
33559 ΔOXA/pRY-S91A-OXA	*bla*_OXA-61_::kan/pRY-S91A-aOXA	Amp^r^, Mem^r^, Kan^r^, Cm^r^	This study
33559 ΔOXA/pRY112	*bla*_OXA-61_::kan/pRY112	Kan^r^, Cm^r^	This study
33559 ΔOXA/pRY-eOXA	*bla*_OXA-61_::kan/pRY-eOXA	Mem^r^, Amp^r^, Kan^r^, Cm^r^	This study
*E. coli* SC12	*bla*_NDM-5_	Amp^r^, Mem^r^	Our lab

^*a*^ Compared with the genetic characteristics of the reference strain ATCC 33559.

^*b*^ Mem, meropenem; Amp, ampicillin; Kan, kKanamycin; Cm, chloramphenicol.

^*c*^ Refer to *Campylobacter coli* NCTC 11366 (ATCC 33559) for detailed strain information.

To validate the affinity between meropenem and the drug-binding pocket of OXA-61, two alanine-substituted variants (33559 ΔOXA/pRY-S42A-OXA and 33559 ΔOXA/pRY-S91A-OXA) were constructed in a chromosomal *bla*_OXA-61_ knockout background. Appropriate control strains—33559 ΔOXA/pRY112 (empty vector) and 33559 ΔOXA/pRY-eOXA (promoter-up variant)—were generated in parallel. Gene deletion and plasmid introduction were performed as described in the preceding section. Bacterial plasmids constructed in this study are listed in Table 5.

**TABLE 5:** Bacterial plasmids used in this study for functional characterization

Plasmid	Description	Resistance marker^*a*^	Source
pRY112 (52)	*Escherichia coli-Campylobacter* shuttle plasmid. PRY112 contains a chloramphenicol resistance marker and a multiple cloning site, providing a plasmid backbone for gene expression.	Cm^r^	Our lab
pRY-wOXA	Containing the PRY112 plasmid backbone and wild-type *bla*_OXA-61_ cassette.	Cm^r^	This study
pRY-eOXA	Containing the PRY112 plasmid backbone and expression-enhanced *bla*_OXA-61_ cassette.	Cm^r^	This study
pRY-S42A-OXA	Containing the PRY112 plasmid backbone and alanine-substituted bla_OXA-61_ gene cassette.	Cm^r^	This study
pRY-S91A-aOXA	Containing the PRY112 plasmid backbone and alanine-substituted *bla_OXA-61_* gene cassette.	Cmr	This study
pRRK (51)	*Escherichia coli* plasmid with pGEM backbone. Containing an ampicillin resistance marker, with a plasmid backbone used for the construction of a suicide plasmid for chromosomal editing in *Campylobacter*.	Kan^r^	This study
pGEM-OXA-KAN	Suicide plasmid containing pGEM backbone and expression-enhanced *bla*_OXA-61_ cassette.	Amp^r^	This study
pGEM-MOMP-KAN	Suicide plasmid containing pGEM backbone, *porA* mutant, and kanamycin resistance marker.	Amp^r^, Kan^r^	This study
pGEM-KO-OXA-KAN	Suicide plasmid containing pGEM backbone and kanamycin resistance marker. Used for the knockout of the *bla*_OXA-61_ gene	Amp^r^, Kan^r^	This study

^*a*^ Amp, ampicillin; Kan, kanamycin; Cm, chloramphenicol.

### Real-time quantitative RT-PCR

*C. coli* strains were cultured on MHA plates and harvested after 12 hours of growth. The OD_600_ of each culture was adjusted to approximately 0.8. Cell pellet was collected by centrifugation at 5,000 × *g* for 10 minutes, resuspended in 100 μL of lysozyme solution (0.5 mg/mL lysozyme, 20 mM Tris-HCl, 2 mM EDTA, pH 8.0), and gently mixed, followed by incubation at room temperature for 5 minutes. Total RNA was extracted using the Takara RNAiso Plus kit. Genomic DNA was removed with gDNA Eraser, and reverse transcription was performed using 1 μg of total RNA per 20 μL reaction system. Real-time PCR was carried out using TB Green Premix with SYBR Green chemistry. The relative expression level of *bla*_OXA-61_ was normalized to that of 16S ribosomal RNA and calculated by the 2^−ΔΔCT^ method. Three independent biological replicates were performed for each strain.

### Prediction of MOMP trimer structure using AlphaFold2

After removal of the signal peptide, the mature MOMP sequence from *C. coli* ATCC 33559 was modeled as a trimer using ColabFold v1.5.5: AlphaFold2 with MMseqs2 (31, 53, 54). Prediction was performed under the adjusted complex mode (24). Multiple loop regions within MOMP protrude toward the exterior of the cell and display considerable flexibility and structural disorder. To better sample alternative conformations of flexible loops, we employed a strategy that restricts the depth of the input multiple sequence alignment in the absence of templates (28, 53). For both the wild-type MOMP and its variants, AlphaFold2 generated 30 trimeric structures, which were then ranked using a weighted scoring function combining the predicted TM-score (pTM) and interface pTM (ipTM): 0.2 × pTM + 0.8 ×ipTM (53).

### Structural analysis of monomeric channels of the mutant MOMP

In PCA) and TM score analyses, these trimers were split, and the loop structures in each individual MOMP monomer were independently evaluated (55). The PCA was performed using Amber-CPPTRAJ (56). TM-align was used to calculate the TM-score between each MOMP monomer structure and the crystal structure (PDB ID: 5LDT). Channel identification and radius calculations were performed with HOLE2 (27), and structural visualization was conducted using PyMOL and VMD. The electrostatic and solvation properties of the channel were calculated using PDB2PQR and Adaptive Poisson-Boltzmann Solver (APBS) (30), and the axial electric field at sampling points along the channel axis was computed using the central difference method. Amino acid sequence and secondary structure alignment of wild-type MOMP (wtMOMP), carbapenem-resistant MOMP (crMOMP), and *C. jejuni* 85H MOMP generated by the ESPript program (57). The stereochemical quality of all primary predicted structures in this study was assessed by PROCHECK (25, 26).

### Molecular dynamics simulations

Conventional MD, SMD, and REUS calculations were performed using GROMACS version 2024.3 (58), patched with PLUMED and compiled with MPICH and CUDA support (59). The trimeric structure of MOMP predicted by AlphaFold2 was aligned to the MOMP crystal structure (PDB ID: 5LDT). Molecular structure files for meropenem (PubChem CID: 441130) and ampicillin (PubChem CID: 6249) were obtained from PubChem. The protonation states of meropenem and ampicillin were optimized using the program Dimorphite-DL (32). The Ca^2+^-binding sites were predicted and positioned using MIB2 (60). A brief energy minimization (1 ns) and equilibration (1 ns) were performed to properly position the coordinating residues. Using the CHARMM-GUI server, standard POPC (1-palmitoyl-2-oleoyl-sn-glycero-3-phosphocholine) lipid bilayer systems for the monomeric channel structure with or without meropenem complex were constructed and then solvated in TIP3P water with 0.15 M NaCl (pH = 7), resulting in systems containing more than 290,000 atoms in total (61–64). Biomolecular components were modeled with the CHARMM36 force field, and the drug was parameterized using the CHARMM General Force Field (CGenFF) (65, 66). Prior to the production MD simulation, the system underwent a four-step pre-equilibration protocol: energy minimization (using the steepest descent algorithm), followed by two consecutive NVT (canonical ensemble) ensemble equilibration phases (with gradual temperature coupling), and a final NPT (isothermal–isobaric ensemble) ensemble equilibration phase to stabilize pressure. The production simulation was then carried out with an integration time step of 2 fs for a total duration of 200 ns. Subsequent analyses, including the calculation of RMSD, RMSF, distance measurements, hydrogen bond statistics, SASA, and Rg, were all performed using the GROMACS tools.

Following the four-step pre-equilibration protocol described above, production molecular dynamics simulations of 200 ns each were carried out for one wtMOMP-membrane system and three crMOMP-membrane systems. Each system underwent three independent simulation replicates. The Ca²^+^-binding pockets were defined as residues 112–126, 149–159, and 314–322.

### SMD and REUS simulations

To obtain the initial configuration for each umbrella window, SMD simulations were performed using the pull module in GROMACS with a direction-periodic pulling method (67). A harmonic potential was applied between the centers of mass of the meropenem molecule and the porin channel exit. The molecule was pulled along the unit vector corresponding to the center axis of the channel, moving from the extracellular side toward the periplasmic chamber and through the channel into the wider cavity on the opposite side. The total pulling time was 4 ns, with system coordinates output every 1 ps. The pulling speed was set to 0.00125 nm/ps, and the force constant was 1,000 kJ/(mol.nm²) to prevent the meropenem molecule from crossing the periodic boundary. Two independent SMD simulations were performed, and suitable frame structures from these trajectories were distributed across the sampling windows as starting points for subsequent REUS calculations (67, 68). Prior to formal REUS production, each window underwent biased processing, including full-energy minimization and equilibration using the same parameter settings as described above. All windows were assigned a force constant of 3,000 kJ/(mol.nm²) to ensure sufficient overlap between windows. Ultimately, exchange probabilities between all adjacent replicas were approximately 20%–30%. BayesWHAM was used to estimate the free-energy profile from the REUS simulations (69).

### Periplasmic content extraction and UPLC analysis

To investigate whether OXA-61 exhibits hydrolytic activity toward meropenem, periplasmic contents were extracted from bacterial cells and incubated separately with meropenem and ampicillin (as a control) (70). The reaction mixtures were monitored using a UPLC method (71, 72).

Bacterial cultures in the logarithmic growth phase (100 μL) were spread onto antibiotic-free MHA plates and incubated for 24 hours. The cells were then harvested, and each bacterial suspension sample was adjusted to an OD_600_ of 1.4. The bacterial cells were collected by centrifugation, and the supernatant was discarded. The cell pellets were resuspended in 1 mL of lysis buffer, which contained 20% sucrose, 3% Tris-HCl solution (1 M, pH 8.0), 0.2% EDTA solution (0.5 M, pH 8.0), and 0.5 mg/mL lysozyme. The mixture of lysis buffer and cells was gently inverted every 10 minutes during lysozyme treatment. After this treatment, 9 mL of ddH_2_O was added, and the reaction was mixed by gentle inversion and incubated on ice for 10 minutes to allow complete release of periplasmic contents. The mixture was centrifuged at 12,000 × *g* at 4°C for 15 minutes, and the supernatant was collected as the periplasmic extract. The periplasmic extract was then used to dilute antibiotic stock solutions to a final concentration of 20 mg/mL for each antibiotic. After mixing the periplasmic extract with the antibiotic solution, the mixture was immediately incubated at 37°C throughout the assay.

Chromatographic analysis was performed on a Thermo UltiMate 3000 UHPLC system equipped with a Welch Xtimate C18 column (2.1 mm × 100 mm; 3 μm). The mobile phases consisted of (A) 0.05% glacial acetic acid, 10 mM potassium dihydrogen phosphate, and 5% acetonitrile in water and (B) 0.05% glacial acetic acid, 10 mM potassium dihydrogen phosphate, and 40% acetonitrile in water. Due to the strong degradation activity of the periplasmic extract toward ampicillin, its elution program was optimized to shorten the detection time and minimize intergroup variation. Therefore, the gradient elution programs for each antibiotic were as follows: for ampicillin: 95% A and 5% B for 1.5 minutes, then 100% A for 1 minute, followed by 95% A and 5% B for 1 minute at a flow rate of 0.5 mL/minute; for meropenem: 85% A and 15% B for 2.2 minutes, then 100% A for 2.8 minutes, followed by 95% A and 5% B for 2 minutes at a flow rate of 0.4 mL/minute. The injection volume was 1 μL, and the detection wavelength was 220 nm for both drugs. The sample compartment was maintained at 37°C, and the column temperature was set to 35°C.

### Molecular docking and binding free energy calculations

The structure of the OXA-61 β-lactamase was predicted using AlphaFold2 with the default parameters, and the highest-scoring conformation was selected for subsequent molecular docking (31, 53). Molecular docking between β-lactamase OXA-61 and the drug was performed using AutoDock Vina (73, 74). Both reasonable β-lactam binding features and optimal binding scores served as dual criteria for the selection of docking structures. Solvent systems were generated using CHARMM-GUI, each containing TIP3P water molecules and appropriate amounts of sodium ions and chloride ions (62). Each system was subjected to the four-step pre-equilibration protocol described above, followed by a 100 ns molecular dynamics production simulation. The last 1,000 frames from each equilibrated simulation trajectory were extracted for subsequent calculation of the binding free energy (ΔG) via the MM/PBSA method (33–35). RMSD, RMSF, free energy landscapes, SASA, and Rg were calculated using GROMACS, and the free energy landscapes were constructed from the last 50 ns of the simulation trajectory. The 2D ligand–protein interaction diagrams were generated using LigPlot + v.2.3 (75).

### Statistical analysis

All statistical analyses were performed using GraphPad Prism 9.5.0. For multiple group comparisons, two-way ANOVA with Dunnett’s multiple comparison test was applied to determine statistical significance, whereas the Kolmogorov–Smirnov test was used to assess distributional differences between two groups. Statistical significance is indicated with asterisks: *P < 0.05; **P < 0.01; ***P < 0.001.

## SUPPLEMENTAL MATERIAL

Supplemental figures and table mbio.00364-26-s0001.docx Fig. S1 to S13 and Table S1.

ASM does not own the copyrights to Supplemental Material that may be linked to, or accessed through, an article. The authors have granted ASM a non-exclusive, world-wide license to publish the Supplemental Material files. Please contact the corresponding author directly for reuse.
