# Supplementary material for: The synergistic action of reduced membrane permeability and antibiotic sequestration as a novel mechanism for carbapenem resistance in Campylobacter
Source: mBio. 2026 May 20;17(6):e00364-26. doi: 10.1128/mbio.00364-26 (PMC13251398; doi:10.1128/mbio.00364-26)
Supplement: Supplemental figures and table — Fig. S1 to S13 and Table S1. [file mbio.00364-26-s0001.docx]

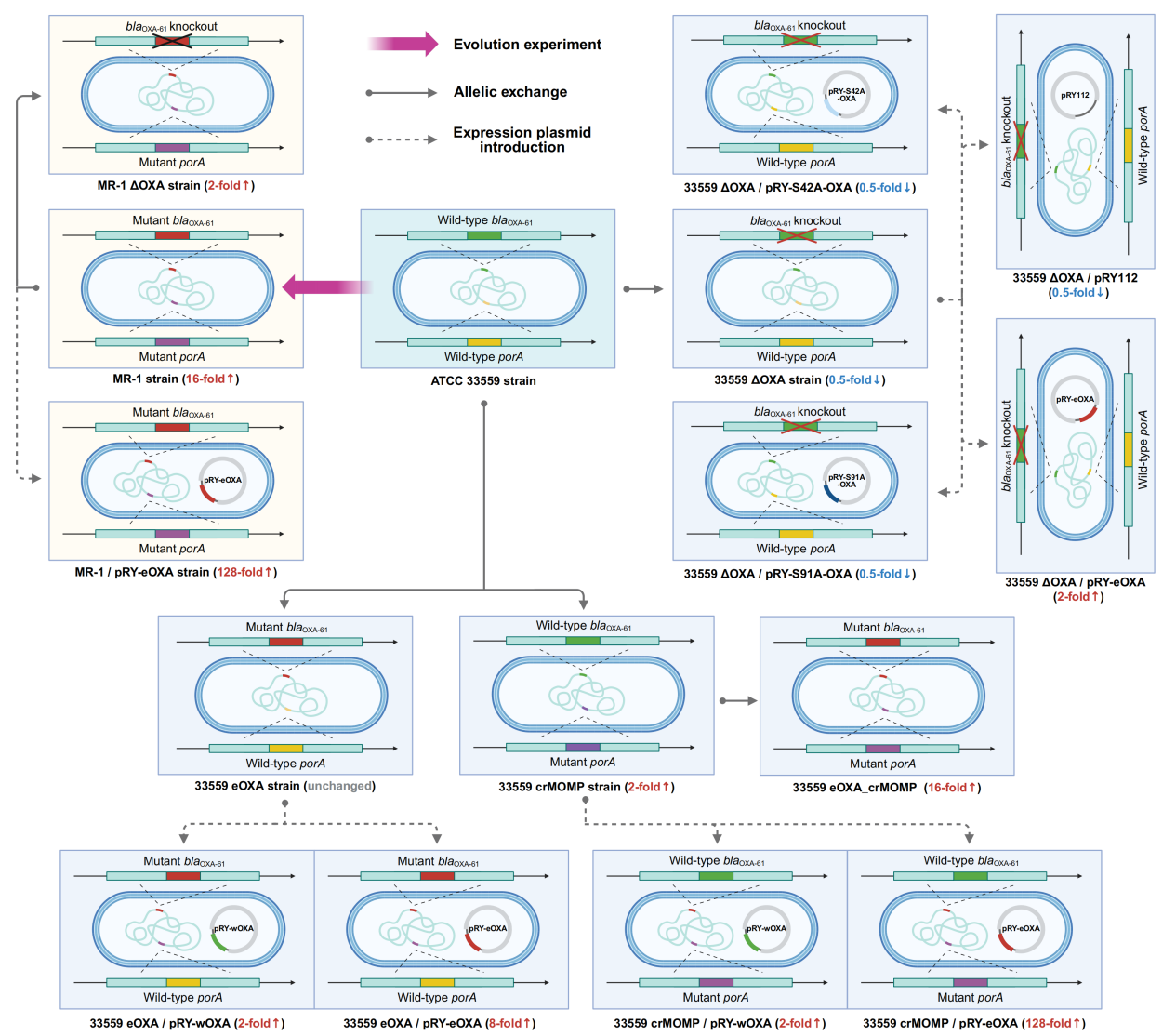


**Figure S1**: **Schematic overview of genetic engineering of lineages used in this study.**

The strains constructed in this study (strain name, genotypes and construction procedures) were used to functionally validate the two mutations. The fold change in meropenem MIC for each strain, relative to the wild-type control (ATCC 33559), is indicated adjacent to the corresponding strain name. The purple solid arrow indicates the derivation of mutant strains via laboratory evolution. Black solid arrows indicate strain construction through suicide plasmid-based allelic exchange. Black dashed arrows indicate the introduction of an expression plasmid harboring frame of *bla*_OXA-61_ or its variants.

**Figure S2**: **Relative expression levels of the *bla*_OXA-61_ gene in different constructs.**

Gene expression was determined by RT-qPCR using *C. coli* ATCC 33559 as the baseline and 16S ribosomal RNA for normalization. No *bla*_OXA-61_ expression was detected in the 33559 ΔOXA strain or MR-1 ΔOXA strain (not plotted). Data represent the mean ± SD of three independent experiments. One-way ANOVA followed by Dunnett’s multiple comparisons test was performed to determine the statistical significance. Statistical significance is indicated with asterisks: *P < 0.05; **P < 0.01; ***P < 0.001
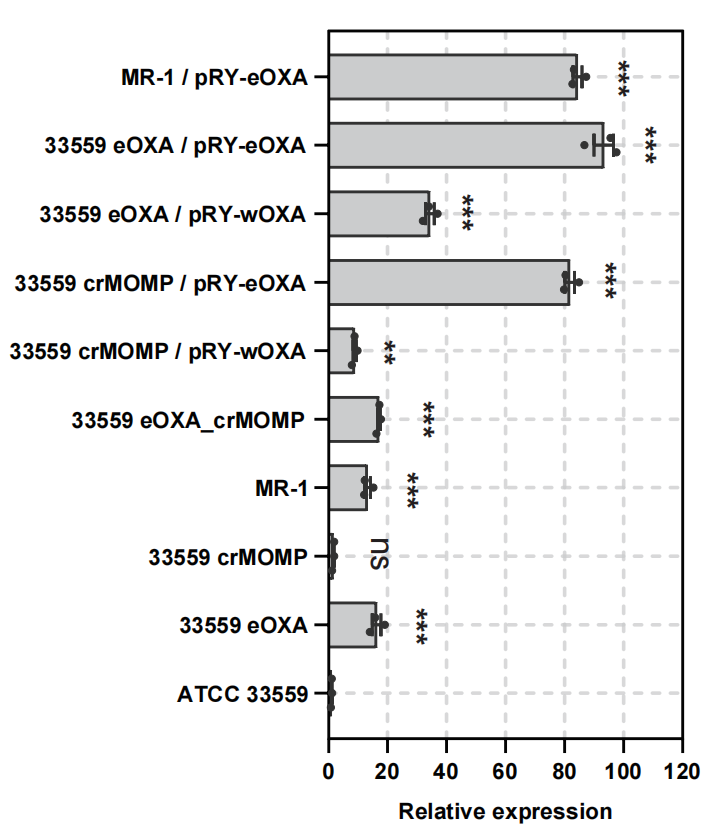
.


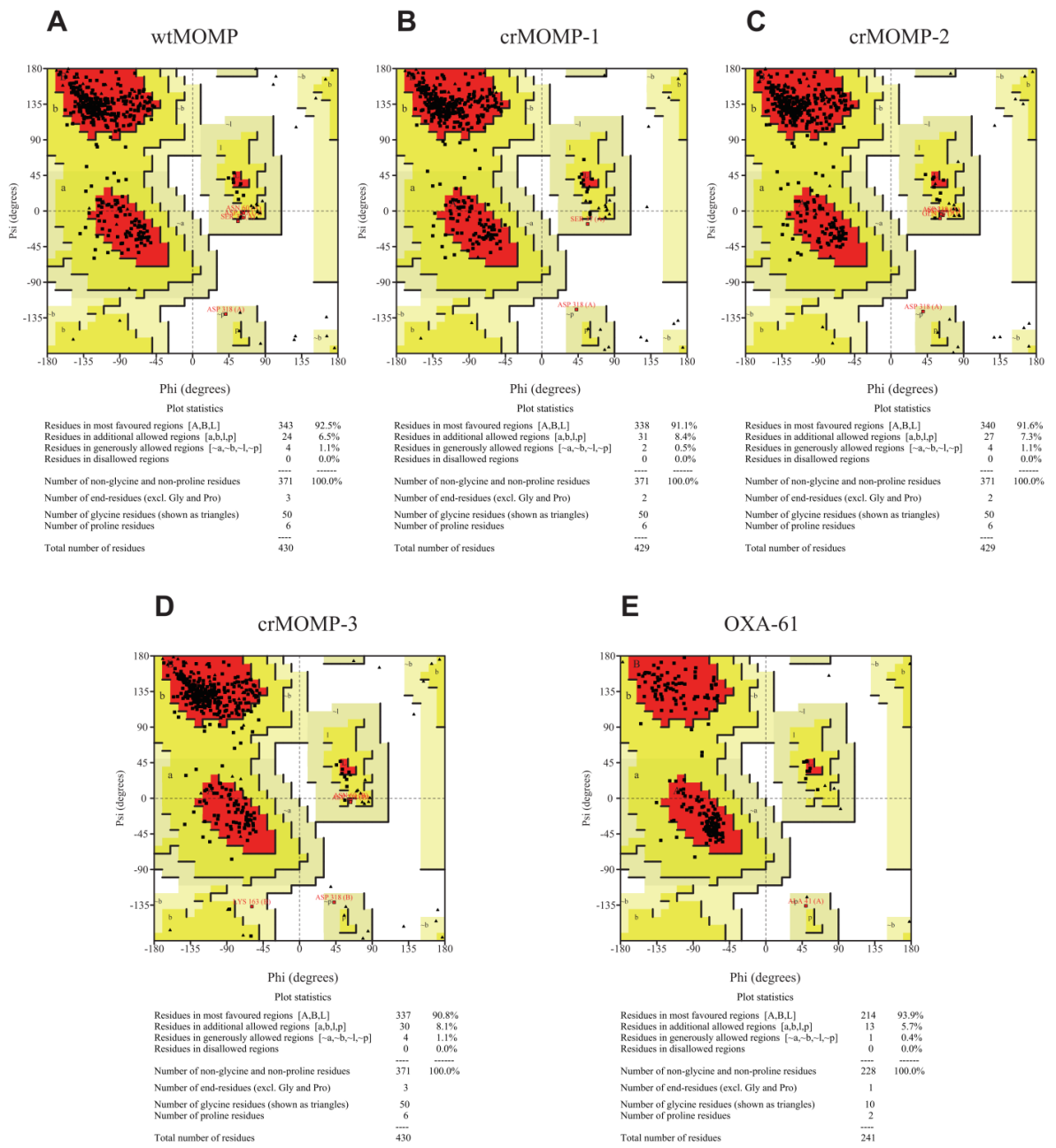


**Figure S3**: Ramachandran plot of the primary predicted structures.

**
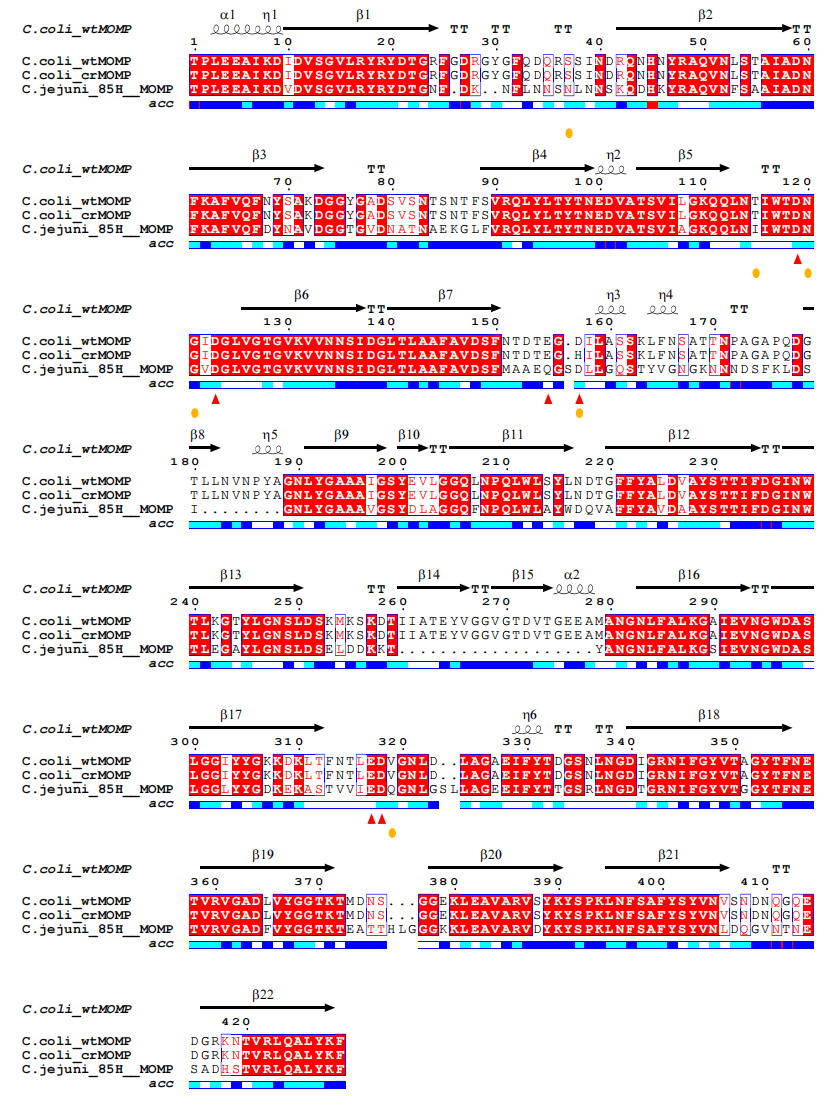
**

**Figure S4: Amino acid sequence alignment of wtMOMP, crMOMP, and *C. jejuni* 85H MOMP.** Residue relative accessibility is indicated as: blue (accessible), cyan (intermediate), and white (buried). Red triangles indicate Ca^2+^-coordinating residues, which are conserved across wtMOMP, crMOMP, and *C. jejuni* 85H. Orange dots indicate mutation sites previously reported to be potentially associated with carbapenem resistance. These mutation sites are primarily located within the Ca^2+^-coordination tunnel.

**
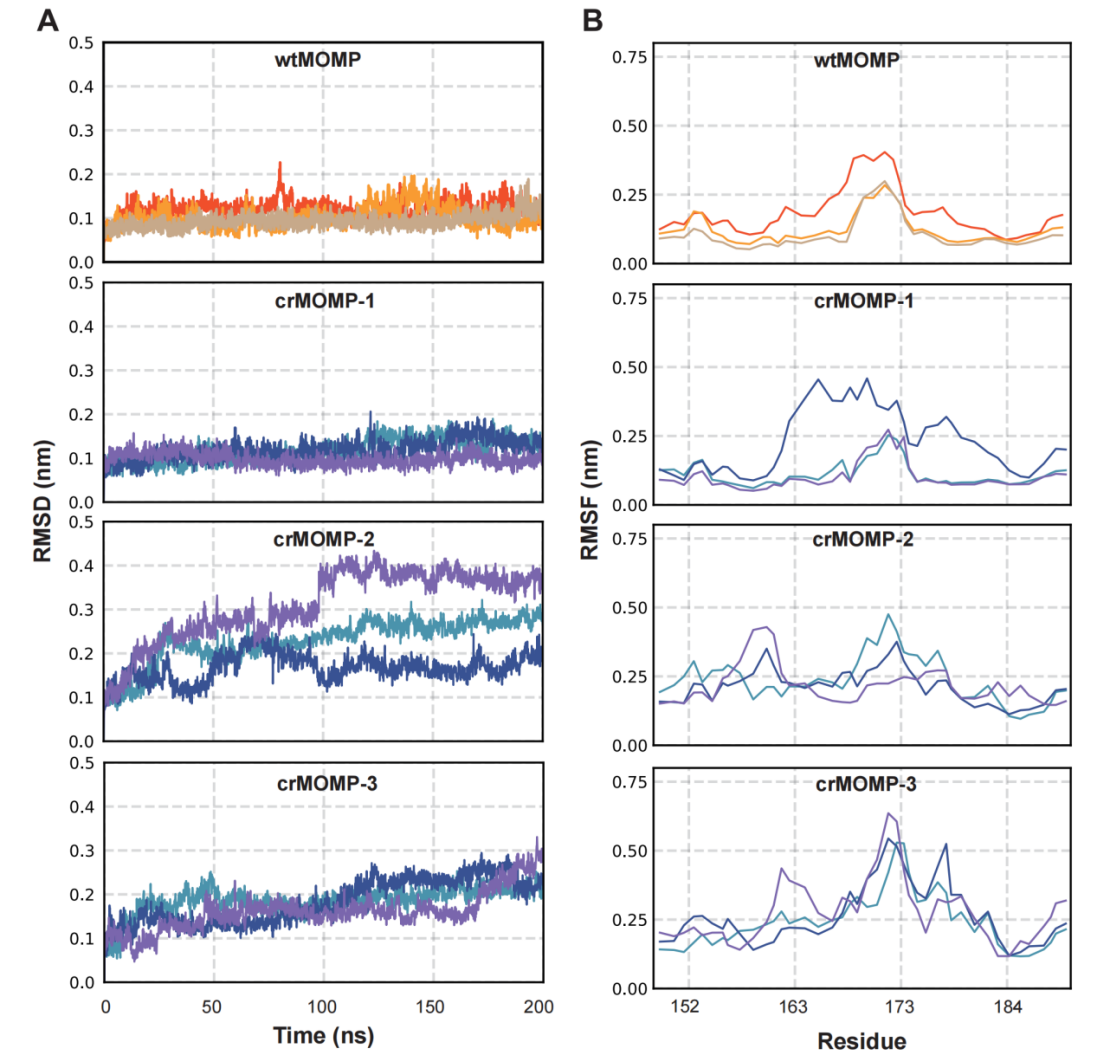
**

**Figure S5: RMSD and RMSF of** **Ca^2+^-binding pocket in MD simulations.** **(A)** The RMSD curves of the Ca^2+^-binding pocket over the 200 ns simulations. **(B)** The RMSD curves of Loop L4 over the 200 ns simulations. For each MOMP conformation, three independent MD production were performed for RMSD and RMSF calculations.

**
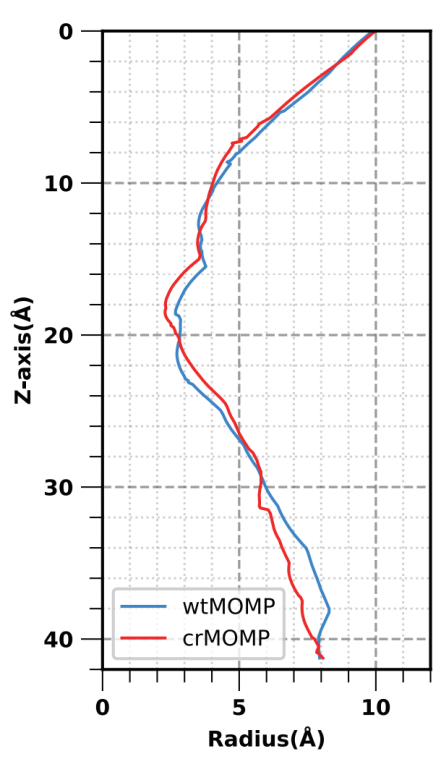
Figure S6：The initial pore radii of wtMOMP and crMOMP.**

**
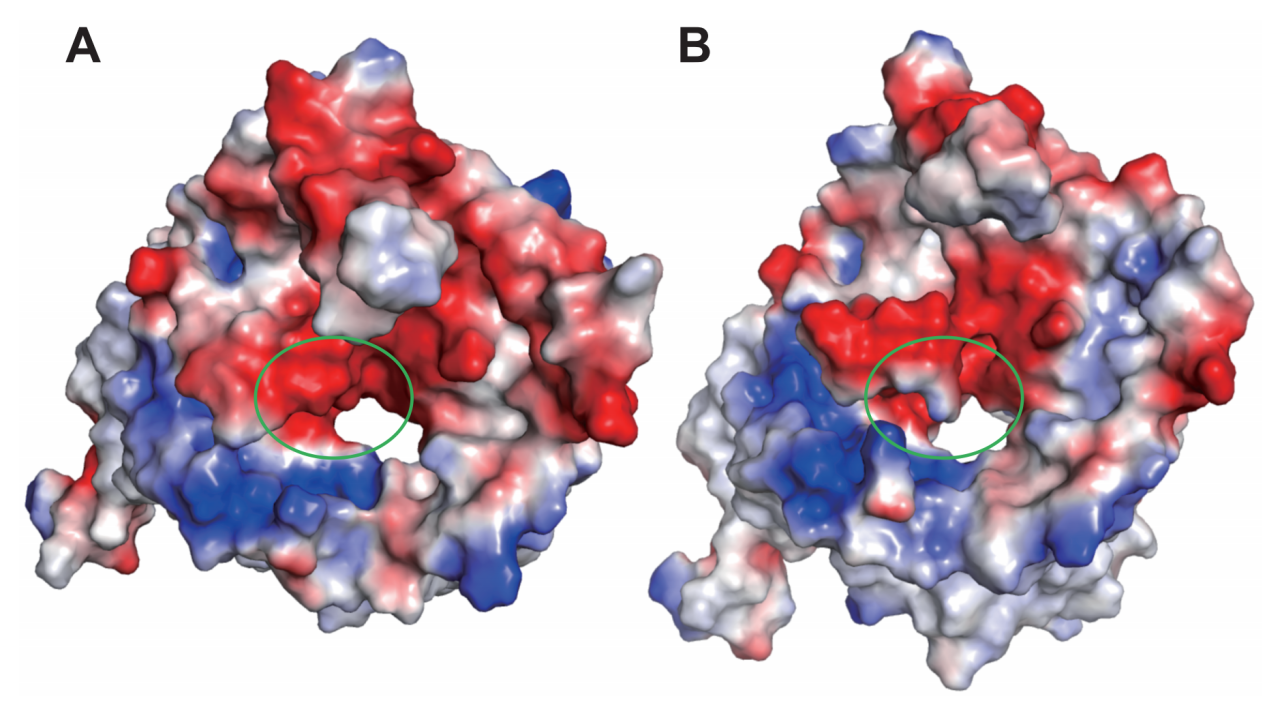
**

**Figure S7: Surface electrostatic potential of wtMOMP (A) and crMOMP (B).** Residue-157 is marked with a green circle in both models. The D157H mutation reduces the number of negatively charged residues in the eyelet region. Negative and positive electrostatic potentials are colored red and blue, respectively.

**
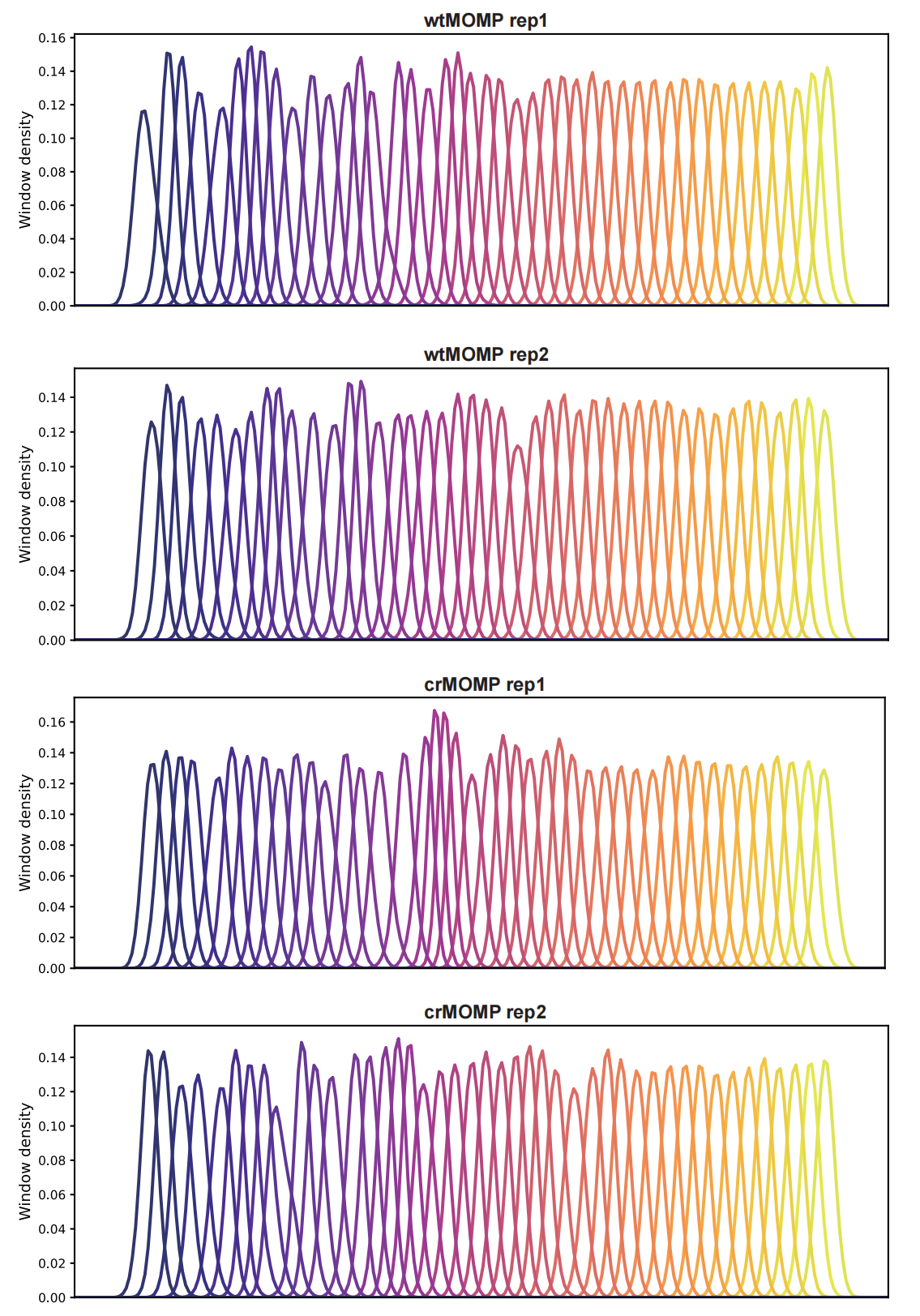
Figure S8: Histograms of the reaction coordinate for the 44 windows.** Windows adjacent to the yellow region indicate that meropenem is situated on the outer side of MOMP, whereas windows adjacent to the blue region indicate that meropenem resides within the interior of MOMP.

**
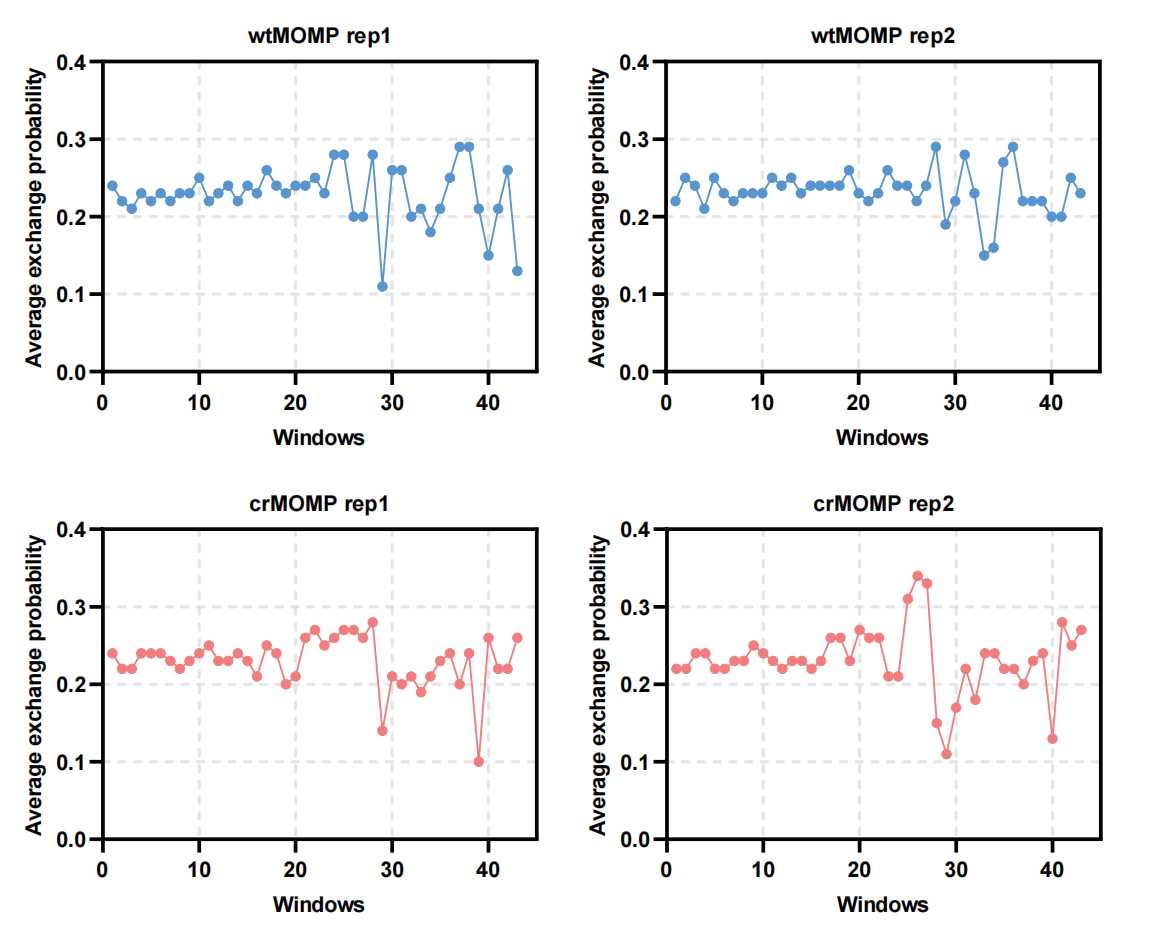
Figure S9: The average exchange probability among 44 windows.**

**Figure S10: 2D ligand-protein interaction diagrams.** The drug and OXA-61 side chains are shown in ball-and-stick representation, with the drug bonds colored in purple. Hydrogen bonds are shown as green dashed lines, and non-bonded interactions between the drug and protein residues are represented by spoked arcs.


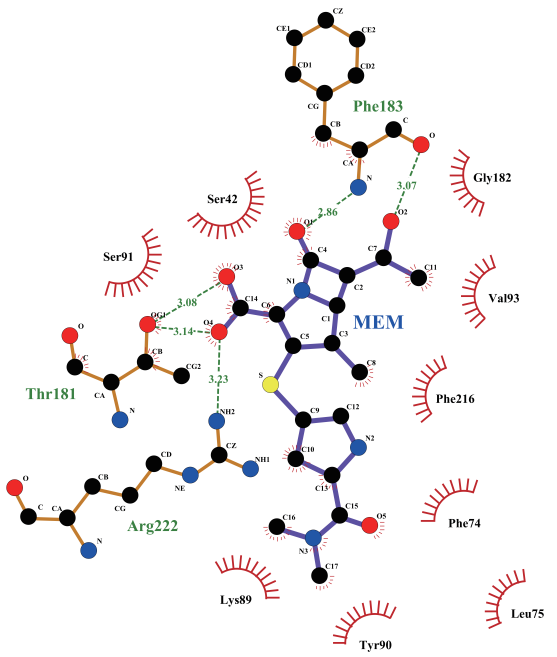

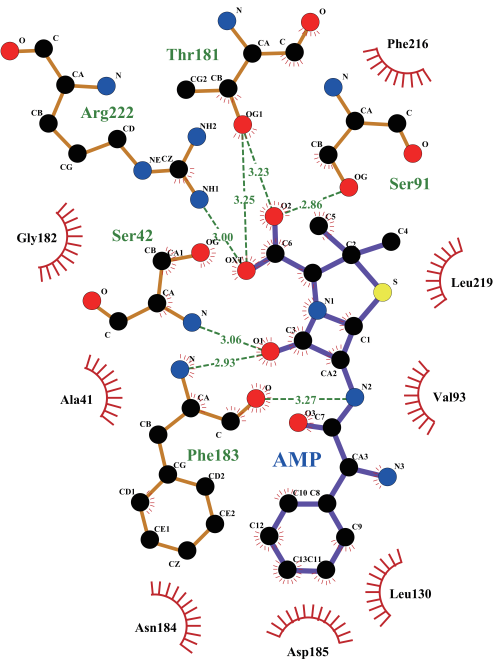


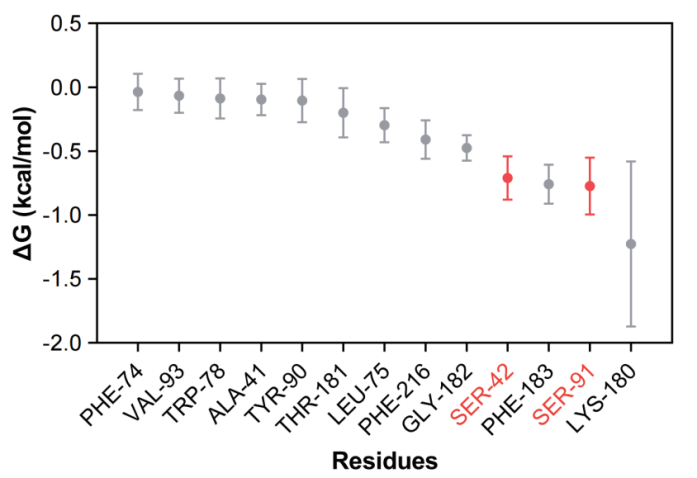


**Figure S11: Ranking of binding free energy contributions from individual residues in the OXA-61 drug-binding pocket.** The two serine residues in the drug binding pocket are marked in red. Both residues are deeply buried at the bottom of the pocket.


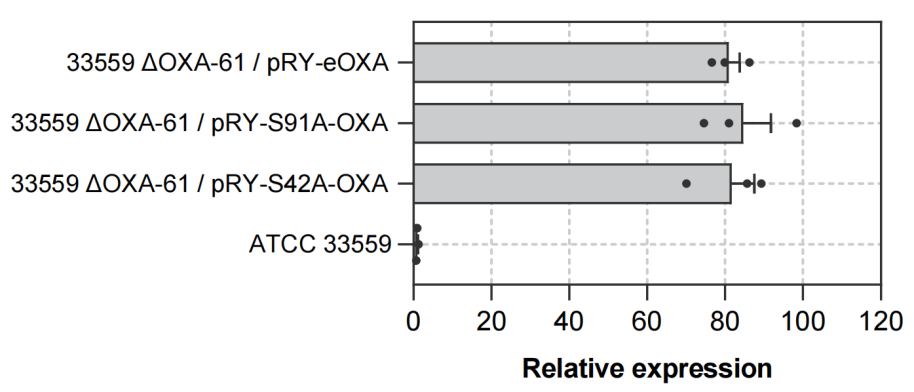


**Figure S12: Relative gene expression levels of *bla*_OXA-61_ alanine mutants.** No *bla*_OXA-61_ expression was detected in the 33559 ΔOXA / pRY112 strain (not plotted). Data represent the mean ± SD of three independent replicates. One-way ANOVA followed by Dunnett’s multiple comparisons test was performed to determine the statistical significance. Statistical significance is indicated with asterisks: *P < 0.05; **P < 0.01; ***P < 0.001.


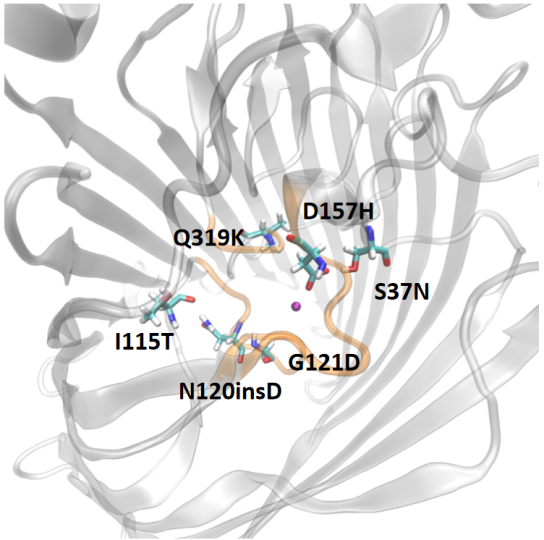


**Figure S13: Locations of mutations reported to be potentially associated with carbapenem resistance in *Campylobacter*.** Orange highlights indicate the loop segments that constitute the Ca^2+^-binding pocket. Purple spheres represent Ca^2+^.

| Table S1. List of primers used in this study | | |
| --- | --- | --- |
|  |  |  |
| Name | Purpose | Sequence |
| OXA_hr_F | cloning *bla*_OXA-61_ (G-T) and construct pGEM-OXA | TATAGGGCGAATTGGGCCCGACGTCTTGTCAAGCCAAAAAGTATCG |
| OXA_hr_R |  | TGCATCCAACGCGTTGGGAGCTCTTGCTTGCGATTTCTATACCC |
| PORA_hr_1 | cloning *porA* and construct pGEM-MOMP-KAN | ATGCATCCAACGCGTATGAAGAGCTTAAGGATAGTGTAGA |
| PORA_hr_2 |  | GGGAATTCGATTTGGTTGCTGCAGGTGCTTTCTC |
| PORA_hr_3 |  | AATTTTCTTCAAGCTCCACAATGATAGAACCAACGATAA |
| PORA_hr_4 |  | CCAAGCTAAAAAGCTAGGCAGCGCGCTTATCAA |
| KO_1 | construct pGEM-KO-OXA-KAN | TGGATAACCGTATTAACATAATCAAGCCTAGCTTCTG |
| KO_2 |  | GTGCCGCTTGTATTATACTC |
| KO_3 |  | TAATACAAGCGGCACAGGCAGCGCGCTTATCAA |
| KO_4 |  | CCTACAATCCAAGCACCACAATGATAGAACCAACGATAA |
| KO_5 |  | TGCTTGGATTGTAGGTTTTGTG |
| KO_6 |  | TGAATTGTAATACGAAAGAACACGAACTTGGCTTTAC |
| pRRK_F | cloning pGEM backbone | TAATACGGTTATCCACAG |
| pRRK_R |  | TCGTATTACAATTCACT |
| PYR_F | cloning pRY112 backbone | GACGTTGGAGTCCACGTTC |
| PYR_R |  | CTTGGCGTAATCATGGTC |
| OXA_OE_F | cloning *bla*_OXA-61_ or *bla*_OXA-61_(G-T). Construct pRY-wOXA and pRY-eOXA. | CATGATTACGCCAAGTCGATGGATTGCTTTAATGGTCACA |
| OXA_OE_R |  | GTGGACTCCAACGTCCGCTTAAGTGCAACTTTGCTAGAAA |
| OXA_seq_F | Validation of transformants | GTGCCGCTTGTATTATACTC |
| OXA_seq_R |  | ATACGCACTTACCATGG |
| PORA_seq_F |  | CCACAAGCGTAATCCTAGG |
| PORA_seq_R |  | GTTCCTACTCCGCCAAC |
| qOXA_F | RT-qPCR | ATTCATATCTTGCGCCCAAG |
| qOXA_R |  | GCTCTAATTGCACTTGATAGTGG |
| q16S_F | RT-qPCR (internal control) | TACCTGGGCTTGATATCCTA |
| q16S_R |  | GGACTTAACCCAACATCTCA |
| S91A_F1 | Construct pRY-S91A-OXA | GTTCAGCTATAAAATATGCTAATGTTCTTGC |
| S91A_R1 |  | CTATCCACTTTTCAATCTATATCACGC |
| S91A_F2 |  | TTGAAAAGTGGATAGATTTATGATATAGTGG |
| S91A_R2 |  | ATTTTATAGCTGAACTTAAATTCATATCTTGCG |
| S42A_F1 | Construct pRY-S42A-OXA | CTTTCTCTCCCGCTGCTACTTTTA |
| S42A_R1 |  | CTATCCACTTTTCAATCTATATCACGC |
| S42A_F2 |  | TTGAAAAGTGGATAGATTTATGATATAGTGG |
| S42A_R2 |  | GTAGCAGCGGGAGAGAAAGTC |
